# Primary healthcare expansion and mortality in Brazil’s urban poor: A cohort analysis of 1.2 million adults

**DOI:** 10.1371/journal.pmed.1003357

**Published:** 2020-10-30

**Authors:** Thomas Hone, Valeria Saraceni, Claudia Medina Coeli, Anete Trajman, Davide Rasella, Christopher Millett, Betina Durovni

**Affiliations:** 1 Public Health Policy Evaluation Unit, School of Public Health, Imperial College London, London, United Kingdom; 2 Health Surveillance Branch, Secretaria Municipal de Saúde do Rio de Janeiro, Rio de Janeiro, Brazil; 3 Instituto de Estudos em Saúde Coletiva, Universidade Federal do Rio de Janeiro, Rio de Janeiro, Brazil; 4 Programa de Pós-graduação em Clínica Médica, Federal University of Rio de Janeiro, Rio de Janeiro, Brazil; 5 Mestrado Profissional em Atenção Primária à Saúde, Federal University of Rio de Janeiro, Rio de Janeiro, Brazil; 6 Instituto de Saúde Coletiva, Universidade Federal da Bahia, Salvador, Brazil; 7 Department of Preventive Medicine, School of Medicine, University of São Paulo, São Paulo, Brazil; 8 Center of Data and Knowledge Integration for Health (CIDACS), Instituto Gonçalo Muniz, Fundação Oswaldo Cruz, Salvador, Brazil; 9 Centro de Estudos Estratégicos, Fundação Oswaldo Cruz, Rio de Janeiro, Brazil; University of California, Los Angeles, UNITED STATES

## Abstract

**Background:**

Expanding delivery of primary healthcare to urban poor populations is a priority in many low- and middle-income countries. This remains a key challenge in Brazil despite expansion of the country’s internationally recognized Family Health Strategy (FHS) over the past two decades. This study evaluates the impact of an ambitious program to rapidly expand FHS coverage in the city of Rio de Janeiro, Brazil, since 2008.

**Methods and findings:**

A cohort of 1,241,351 low-income adults (observed January 2010–December 2016; total person-years 6,498,607) with linked FHS utilization and mortality records was analyzed using flexible parametric survival models. Time-to-death from all-causes and selected causes were estimated for FHS users and nonusers. Models employed inverse probability treatment weighting and regression adjustment (IPTW-RA).

The cohort was 61% female (751,895) and had a mean age of 36 years (standard deviation 16.4). Only 18,721 individuals (1.5%) had higher education, whereas 102,899 (8%) had no formal education. Two thirds of individuals (827,250; 67%) were in receipt of conditional cash transfers (*Bolsa Família*). A total of 34,091 deaths were analyzed, of which 8,765 (26%) were due to cardiovascular disease; 5,777 (17%) were due to neoplasms; 5,683 (17%) were due to external causes; 3,152 (9%) were due to respiratory diseases; and 3,115 (9%) were due to infectious and parasitic diseases. One third of the cohort (467,155; 37.6%) used FHS services. In IPTW-RA survival analysis, an average FHS user had a 44% lower hazard of all-cause mortality (HR: 0.56, 95% CI 0.54–0.59, *p* < 0.001) and a 5-year risk reduction of 8.3 per 1,000 (95% CI 7.8–8.9, *p* < 0.001) compared with a non-FHS user. There were greater reductions in the risk of death for FHS users who were black (HR 0.50, 95% CI 0.46–0.54, *p* < 0.001) or *pardo* (HR 0.57, 95% CI 0.54–0.60, *p* < 0.001) compared with white (HR 0.59, 95% CI 0.56–0.63, *p* < 0.001); had lower educational attainment (HR 0.50, 95% CI 0.46–0.55, *p* < 0.001) for those with no education compared to no significant association for those with higher education (*p* = 0.758); or were in receipt of conditional cash transfers (*Bolsa Família*) (HR 0.51, 95% CI 0.49–0.54, *p* < 0.001) compared with nonrecipients (HR 0.63, 95% CI 0.60–0.67, *p* < 0.001).

Key limitations in this study are potential unobserved confounding through selection into the program and linkage errors, although analytical approaches have minimized the potential for bias.

**Conclusions:**

FHS utilization in urban poor populations in Brazil was associated with a lower risk of death, with greater reductions among more deprived race/ethnic and socioeconomic groups. Increased investment in primary healthcare is likely to improve health and reduce health inequalities in urban poor populations globally.

## Abstract 2

Thomas Hone and colleagues explore the Family Health Strategy for primary health care access in a low-income population.

## Author summary

**Why was this study done?:**

- There is evidence, predominantly from high-income countries, that health systems with stronger primary healthcare (PHC) have better health outcomes, reduced health inequalities, and reduced financial impoverishment from healthcare costs.
- However, evidence from low- and middle-income countries (LMICs) often has methodological weaknesses, focuses heavily on child and maternal health, does not focus on vulnerable groups such as the urban poor, and is not often disaggregated by socioeconomic groups.
- The city of Rio de Janeiro has undergone a major expansion of PHC services since 2008, with investments in electronic medical records that provide a valuable experimental setting for evaluating PHC.

**What did the researchers do and find?:**

- Administrative welfare records of 1.2 million low-income adults in the city of Rio de Janeiro were linked with PHC records and death certificates between 2010 and 2016.
- The risk of death for PHC users was compared with nonusers with statistical adjustment differences between groups.
- Compared with nonusers, PHC users had reduced risk of death, but those with lower education, receiving welfare, or who were black or *pardo* (mixed-ethnicity) had the greatest reductions.

**What do these findings mean?:**

- PHC usage is associated with health benefits in low-income urban populations in an LMIC and, with greater benefits in deprived socioeconomic groups, there is the potential to reduce health inequalities.
- Policymakers should continue to prioritize and invest in PHC as part of health system strengthening efforts and actions for progress toward Universal Health Coverage.

## Introduction

Strengthening primary healthcare (PHC) for urban poor populations remains a major global challenge [1]. The urban poor are increasingly burdened by noncommunicable diseases from energy-dense diets and inactive lifestyles, persisting risk of infectious diseases from poor living conditions, and high levels of road traffic accidents, violence, and crime [2]. Yet these populations are often ignored by society, and access to high-quality healthcare is low [1]. Worldwide, one billion poor people live in slums, where barriers to expanding healthcare include poverty (and low ability to pay), hazardous environments, poor infrastructure, violence, and weak political representation [3]. Progress in expanding healthcare to the urban poor is further inhibited by a paucity of evidence examining which models of PHC delivery are acceptable, feasible, and effective in these settings [4] and whether PHC can address social gradients in health among urban poor populations [5].

Brazil is an internationally important setting for examining the impact of PHC in urban poor populations. One fifth of the population (42 million) live on less than $5.50 USD a day (the upper middle-income poverty line), whereas one fifth of the urban population live in slums or *favelas* [6]. This is symbolic of the stark inequalities in a country forecast to achieve less than one third of health-related Sustainable Development Goal targets by 2030 [7]. Although Brazil has made major progress in expanding access to PHC through expansion of the internationally recognized *Estratégia de Saúde da Família* (Family Health Strategy [FHS]) [8], coverage has lagged substantially behind in urban poor communities living in cities including São Paulo, Salvador, and Rio de Janeiro (S1 Fig). The FHS is a model of PHC in which multidisciplinary teams, including community health agents, cover approximately 1,000 local families and provide a range of services including acute care, referral, risk factor management, prevention, health promotion, and health education, and home visits [9]. FHS expansion in Brazil has been associated with reductions in infant mortality [10,11], cardiovascular disease mortality [12,13], amenable mortality [13], and racial health inequalities [14], but the specific health and health inequality impacts in urban poor populations have not been studied.

The city of Rio de Janeiro substantially expanded PHC coverage since 2008 with an ambition to deliver universal access to comprehensive, high-quality care to all residents. The early phases of the program prioritized PHC expansion predominately in poor areas lacking services where unmet health need was greatest. Before FHS expansion, low-income populations in the city, who are often priced out of private healthcare, were largely reliant on overcrowded emergency rooms [15]. This study uses a novel individual-level dataset of 1.2 million low-income adults in the city (covering the poorest 25% of the population) with linked primary care, socioeconomic, welfare claimant, and mortality data to evaluate the program. Survival analysis was used to examine associations between primary care utilization and all-cause and cause-specific mortality. Large individual-level data permit a health equity assessment of program impacts, including between racial groups, which is seldom possible in PHC evaluations globally because of the reliance on ecological or survey datasets [11]. This study assesses the association of PHC usage and mortality of low-income adults and whether there are differences across socioeconomic groups and causes of deaths.

### Expansion of the FHS in Rio de Janeiro

Rio de Janeiro is the second largest city in Brazil and has some of the country’s largest socioeconomic and health inequalities. Over one third of Rio’s population (2.5 million) live in *favelas [16]*. Life expectancy varies by nearly 13 years between wealthy areas and *favelas* [17], with infant mortality rates 5 times higher in *favelas* [18]. Brazilian cities have considerable autonomy in the provision of local healthcare, but historical investment and coverage of PHC have been low in Rio de Janeiro. In 2008, the city had the lowest public expenditure on health of any state capital; over 80% of the city’s health budget was allocated to hospitals, and FHS coverage was 7% [19,20]. Service quality was also low, with 40% of FHS teams lacking doctors [19]. Low-income populations were reliant on hospital and outpatient care or a few private and philanthropic clinics for healthcare [15,21,22].

Poor population health (including high rates of tuberculosis, congenital syphilis, and infant and maternal mortality) and attention on the city’s low FHS coverage encouraged healthcare reform [20]. The 2009 Strategic Plan of the Mayor’s Office committed resources to expand FHS in the city, especially to poor and disadvantaged populations [19]. A strategy for PHC was developed based on fundamental principles of accessibility, continuity, coordination, and integration, drawing on international examples of PHC (including Portugal and the United Kingdom) [4,19,23]. The Secretariat for Health was restructured around delivering this strategy. This involved quality improvement initiatives, including pay for performance, strengthened clinical governance mechanisms, and investment in electronic medical records. Novel contracting arrangements facilitated higher doctor salaries but with concurrent requirements for specialized training and participation in continuing professional development activities. A family medicine residency program was also introduced (one of the first in the country) to encourage specialization in family medicine [19].

The FHS is Brazil’s main vehicle for achieving universal health coverage (UHC) and centers on multidisciplinary healthcare teams, geographically defined catchment populations, and proactive outreach services provided by community health workers [9]. Services are free to use and cover locally registered populations. The FHS was adapted in Rio de Janeiro to focus on quality, coordination, and efficiency and to meet the needs of urban poor populations who were initially prioritized under expansion [19]. This was enacted through evidence-based protocols, detailed job descriptions, training and workshops, budgetary meetings with FHS teams, and close quality monitoring [19,20]. Multiple FHS teams are co-located in clinics, which are equipped with radiological services, ultrasound, and equipment for minor surgery facilitating comprehensive service provision [24]. The number of FHS teams increased from 128 in 2008 to 855 in 2015 (located in 194 clinics), with over 50% of the Rio de Janeiro population covered in 2016.

## Methods

### Study design

This study is a cohort analysis of 1.2 million low-income adults, residents in the city of Rio de Janeiro, Brazil. The cohort was analyzed longitudinally using survival analysis, which allowed variable periods of observation for individuals and time-varying coverage under FHS. Doubly robust inverse probability treatment weighting and regression adjustment (IPTW-RA) were employed to maximize causal inference through minimizing bias from nonrandom allocation to treatment (FHS use) and differences in between control and treated groups [25].

The broad analytical approach (survival analysis approaches) for this study was devised for the funding proposal (June 2016). Data linkage was completed in November 2018, and further development of analytical approaches began then. Initial data analyses in December 2019 led to further considerations for potential biases relating to potential selection into the FHS, and the need to account for time-varying exposure to the FHS and the desire to compute absolute effect sizes led to the current analytical approach. Following initial results and sensitivity analyses, the analytical approach in the paper was agreed upon in January 2020 before expanding the approach to other outcomes and subgroup analyses.

### Study population and data sources

The study population was adults (aged 15–84 years) registered in the *Cadastro Único* database between 1 January 2010 to 31 December 2014 and living in the municipality (city) of Rio de Janeiro. The *Cadastro Único* is a national administrative database of all individuals claiming government welfare (see S1 Table). The dataset covers roughly 25% of the city population, contains a wide range of individual- and household-level characteristics for both FHS users and nonusers, and is feasible for linkage with healthcare and mortality records. Given the low-income coverage of the *Cadastro Único*, it is likely that most live in favelas.

The *Cadastro Único* records were linked to 3 additional datasets (S1 Table and S1 Text): (1) FHS electronic health records, including individuals’ date of registration and utilization; (2) mortality records for 2010–2016 including date and cause of death (ICD10 code); and (3) public hospitalization records including date of admission. All datasets were linked via a combination of deterministic and probabilistic approaches (S1 Text). This involved matching name, date of birth and *Cadastro de Pessoas Físicas* (CPF) numbers (Brazilian tax numbers) using deterministic linkage, phonetic matching, and Levenstien distance matching. Records were extensively reviewed by multiple investigators to ensure high-quality and accurate matching. Duplicate records, those with invalid registration throughout the period, and those erroneously registered in the *Cadastro Único* after their death were excluded.

### Variables

The primary outcome was all-cause mortality measured in survival analysis as time-to-death (identified from date on death certificates). Deaths from selected groups of causes were secondary outcomes (S2 Table).

The variable of interest (exposure) was FHS use. To identify FHS users, the date of first primary care consultation with a doctor or nurse (any time before December 2016) was used to define the beginning of an individual’s FHS coverage. As many individuals gained FHS coverage partway through the observation period, it was important to adjust for their non-FHS-covered observation time to reduce immortal time or survivor treatment selection biases [26]. This was done by modeling FHS coverage as time-varying, allowing both non-FHS and FHS time periods for an individual to be captured. Individuals could change FHS status once during the cohort, from nonuser to user.

Covariates were included for both modeling individuals’ likelihood of FHS use (IPTW) and adjusting survival analysis models. These were to account for demographic and socioeconomic characteristics (or proxies for these factors), which are indicative of deprivation and underlying health needs. Individual-level covariates were (see S1 Table) sex; race/ethnicity (in Brazil, this is based on self-reported skin color); age; highest level of education; disability; unemployment; formal labor employment; and whether the individual has been hospitalized before FHS use. Household-level covariates were per capita household income decile; family members per bedroom; family size; total children in family; household flooring; household piped water access; quintiles of household expenditure on medicines; quintiles of per capita household expenditure on food; formal labor employment in the family; and whether the family receives *Bolsa Familia* (a conditional cash transfer program) [27]. Collinearity was checked with variance inflation factors (VIFs).

### Analysis

Individuals in the cohort had a start date at any point from 1 January 2010 to 31 December 2014 (when they joined *Cadastro Único* or turned 15 years of age). End dates were defined as an individual’s date of death, or for surviving individuals (who were right-hand censored), their 85th birthday or 31 December 2016. The time scale used for analysis was time-on-study. Multiple steps of analysis were carried out.

Firstly, weights for IPTW were generated using baseline data (S2 Text). Adjusted logistic regression on the likelihood of an individual ever using the FHS (binary outcome) was used to calculate predicted probabilities of FHS use and IPTW [25]. Secondly, data were descriptively analyzed with the unweighted and weighted distributions of covariates presented. Standardized difference measures were used to compare covariate balance between FHS users and nonusers, with a smaller value indicated a more balanced sample [25,28] (S3 Table).

Thirdly, flexible parametric survival analysis was used to model time-to-death. Survival analysis is the most appropriate method for evaluating the association between FHS usage and mortality using time-to-event data and also incorporating the time-varying nature of FHS registration. Flexible parametric survival analysis models were chosen as they have greater flexibility than more traditional survival models permitting time-varying covariates, allow both relative and absolute effect sizes to be estimated, and are suitable for out of observation prediction [29]. A proportional hazard survival distribution was chosen based on the Akaike information criteria (AIC) (S4 Table) [29]. Effect estimates are interpreted similarly to other survival analysis models such as Cox’s proportional hazards, in which hazard ratios (HRs) represent relative differences in hazard rates. The post regression prediction of absolute effect sizes was important, given the small risk of death over the study period. The 5-year risk differences (RDs) between FHS users and nonusers were also estimated from predicted survival function at 5 years and expressed per 1,000 individuals with covariates held at their mean values.

The primary outcome, time until all-cause mortality, was analyzed using these flexible parametric survival models with IPTW-RA. IPTW-RA aimed to reduce potential unmeasured confounding related to an individual’s underlying health needs and the propensity to seek healthcare. The covariates employed represent deprivation and socioeconomic status associated with underlying healthcare needs. HRs and 5-year RD were reported in addition to adjusted survival curves for FHS users and nonusers. Covariates were held at their mean values for these estimates (i.e., the average individual in the cohort) with the effect estimates of FHS usage interpreted relative to the mean length time since first utilization. Secondary outcomes, by causes of death, were analyzed similarly with HRs and 5-year RD reported.

Time-varying and utilization-varying associations of FHS usage were explored. Time-varying differences were examined by modeling each 6-month interval since an individual’s first use of FHS services and estimating separate HRs. Utilization-varying associations were modeled by calculating FHS users’ total consultations over the period 2010–2016 and estimating HRs for usage groups: no FHS use; 1 consultation; 2; 3; 4; 5, 6–7; 8–9; 10–14; 15–19; 20–29; 30–49; or 50 or more consultations. These modeling approaches also accounted for immortal time bias. HRs from these analyses are interpreted relative to non-FHS users.

Fourthly, heterogeneity of the FHS was explored with subgroup analyses by stratification. Separate stratified regression models were undertaken for each socioeconomic group: sex, race/ethnicity, educational attainment, age group, disability status, whether the family received *Bolsa Familia*, and levels of prior hospitalization. All other covariates as noted here previously were included in the models. Additionally, subgroup analyses by groups of causes of death were explored (see S3 Fig and S4 Fig).

Analyses were carried out in Stata 15 MP (StataCorp LLC; https://www.stata.com/) using the stpm2 command for flexible parametric models [29].

### Sensitivity analysis

Alternative model specifications were tested to explore the robustness of the findings. This included parametric survival analysis with a Gompertz distribution, a Cox’s proportional hazard model, the use of chronological age (rather than time-on-study) as a time scale [30], and unweighted analysis (S4 Table, S6 Table and S2 Fig). Alternative specifications of FHS usage and registration were also tested. Stepwise addition of covariates was used to explore model stability (see S5 Table).

### Ethical approval

Approval for this study was obtained from the Brazilian National Commission for Ethics in Research (*Comissão Nacional de Ética em Pesquisa* [CONEP])—number 2.689.528.

The authors had full access to all anonymized databases employed in this analysis. Identifiable datasets for linkage securely were held by coauthor (C. Medina Coeli) for carrying out linkages and the generation of linkage keys to link the anonymized datasets.

## Results

The cohort contained 1,241,351 adults (6,498,607 person-years) aged 15–84 years at any point during the period (1 January 2010 to 31 December 2016) (Table 1). This was obtained from a starting study population of 1,762,905 individuals, of which 83,583 were identified and removed as duplicates; 424,243 were aged under 15 years at the end of the period; 11,510 died before the start of the period; and 2,218 were 85 years or older at the start of the period. The mean observation period was 5.24 years. There were 34,091 deaths over the period, with cardiovascular disease (CVD) (8,765; 25.7%), neoplasms (5,777; 17.0%), external causes (5,683; 16.7%), respiratory diseases (3,152; 9.3%), and infectious and parasitic conditions (3,115; 9.1%) as the most frequent causes of death.

**Table 1:** Characteristics of cohort by FHS usage.

	*N*		IPTW Distribution (%)	
	Non-FHS users	FHS users	Non-FHS users	FHS users
**Individual characteristics**				
Sex				
Male	349,920	139,536	39.5	39.4
Female	424,276	327,619	60.5	60.6
Race/Ethnicity				
White	231,313	135,696	29.7	29.6
Black	133,224	83,359	17.4	17.4
*Parda* (mixed)	390,738	239,043	50.6	50.7
Other	18,921	9,057	2.3	2.3
Age (years)				
15–17	86,792	45,937	10.9	10.7
18–19	55,419	29,742	6.8	6.8
20–22	78,923	40,003	9.4	9.5
23–24	47,931	22,990	5.6	5.7
25–29	89,739	45,284	10.7	10.8
30–34	71,538	41,687	9.0	9.1
35–39	71,519	43,954	9.2	9.3
40–44	64,731	41,320	8.6	8.5
45–49	55,399	37,435	7.6	7.5
50–59	81,072	62,393	11.8	11.7
60–69	43,973	38,660	6.8	6.8
70+	27,160	17,750	3.7	3.6
Education level				
Preschool/Literacy class/None	67,888	35,011	8.3	8.3
Elementary school	466,275	291,595	60.9	61.0
High school	226,173	135,688	29.2	29.2
Higher education	13,860	4,861	1.5	1.5
Disability				
No	749,625	444,861	96.1	96.1
Yes	24,571	22,294	3.9	3.9
Unemployed				
No	592,497	323,488	73.7	73.7
Yes	181,699	143,667	26.3	26.3
Formally employed?				
No	707,065	426,551	91.3	91.3
Yes	67,131	40,604	8.7	8.7
Hospitalizations prior to FHS use				
None	667,193	417,454	87.0	87.3
one	73,030	37,611	9.8	8.6
two or more	33,973	12,090	3.1	4.1
**Household characteristics**				
Income Deciles				
Q1 (poorest)	79,339	39,498	9.5	9.6
Q2	67,763	42,851	8.8	8.9
Q3	69,608	44,790	9.1	9.2
Q4	71,799	45,486	9.4	9.4
Q5	75,528	46,582	9.8	9.8
Q6	75,506	46,291	9.8	9.8
Q7	76,262	47,376	10.0	10.0
Q8	78,365	49,135	10.3	10.3
Q9	83,163	50,223	10.9	10.8
Q10 (richest)	96,863	54,923	12.4	12.3
Family members per bedroom				
2 or fewer	301,533	195,511	40.2	40.1
more than 2, 3, or fewer	193,851	118,202	25.1	25.1
more than 3, 4, or fewer	141,954	81,036	18.0	17.9
greater than 4	136,858	72,406	16.7	16.8
Family size				
Single person	49,666	29,810	6.4	6.4
Two	97,785	64,920	13.2	13.1
Three	156,885	95,329	20.4	20.3
Four	179,238	105,942	23.0	23.0
Five	130,340	76,983	16.7	16.7
Six or more	160,282	94,171	20.3	20.4
Number of children in family				
None	410,473	229,432	51.9	51.6
One	220,056	138,340	28.8	28.9
Two	98,798	67,316	13.3	13.3
Three	32,202	22,935	4.4	4.4
Four or more	12,667	9,132	1.7	1.7
Household flooring material				
Soil	205,758	90,726	23.9	23.9
Cement	133,109	95,085	18.3	18.4
Repurposed wood	15,761	7931	1.9	1.9
Ceramics/tiles	404,860	266,307	54.1	54.1
Other	14,708	7,106	1.8	1.8
Piped water in household?				
No	23,251	11,453	2.8	2.8
Yes	750,945	455,702	97.2	97.2
***Bolsa Familia***–claiming family?				
No	279,001	135,100	33.6	33.4
Yes	495,195	332,055	66.4	66.6
Quintiles of per capita medicine expenditure				
Q1 (least)	625,271	369,007	80.0	80.1
Q2	53,049	37,775	7.3	7.3
Q3	39,559	25,664	5.3	5.3
Q4	28,599	18,677	3.8	3.8
Q5 (most)	27,718	16,032	3.6	3.5
Formal employment in family				
No	607,335	367,275	78.5	78.5
Yes	166,861	99,880	21.5	21.5
Quintiles of per capita food expenditure				
Q1 (least)	178,555	94,117	21.9	21.9
Q2	143,986	95,867	19.2	19.3
Q3	152,954	96,721	20.1	20.1
Q4	153,741	93,247	20.0	19.9
Q5 (most)	144,960	87,203	18.8	18.7
Total individuals	774,196	467,155		
Total families	381,918	272,974		
Person-Years of observation	4,009,856	2,488,751		
Total deaths (any cause)	30,060	4,031		

FHS, family health strategy; IPTW, inverse probability of treatment weighting; IPTW, distribution shows percentage distributions of variables after IPTW have been applied.

By the end of the period (31 December 2016), 59.9% of the cohort (742,911) had registered with FHS services—an increase from 0.001% (only 599 individuals) at the start (1 January 2010). Of these, 467,155 used FHS services (variable of interest), meaning 37.6% of the total cohort were FHS users. The total person-years of FHS users (time since first contact with the FHS) was 1,330,596 (20.5% of total person-years). The mean time since first FHS use was 2.6 years (SD ±1.5 years).

**Fig 1:**
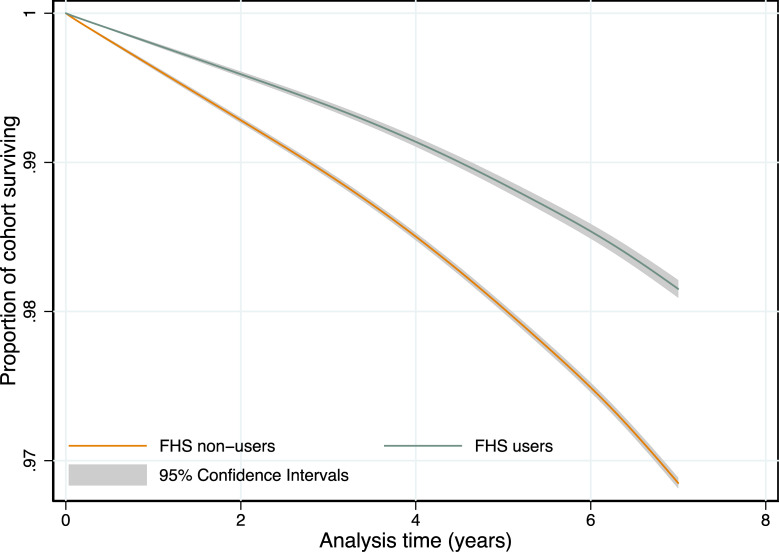
Adjusted survival function of cohort by FHS usage groups. Survival functions obtained from flexible parametric survival models with IPTW and regression adjustment for sex, race/ethnicity, age at cohort entry, highest level of education, disability, unemployment, household per capita income decile, number of family members per bedroom, family size, number of children in family, household flooring, household piped water access, quintiles of household expenditure on medicines, quintile of per capita household expenditure on food, formal labor employment, formal labor employment in the family, whether the family receives *Bolsa Familia* or not, and whether the individual has been hospitalized before FHS use. FHS, Family Health Strategy; IPTW, inverse probability treatment weighting.

**Fig 2:**
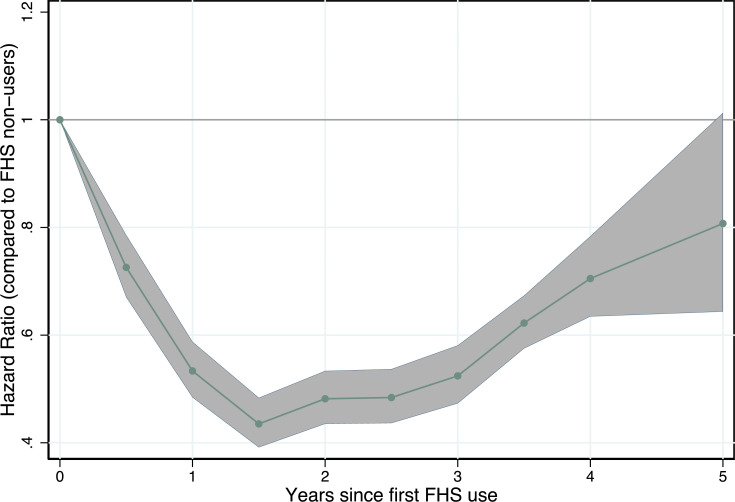
HRs for all-cause mortality by length of time since first usage. Estimated HRs obtained from flexible parametric survival models with IPTW and regression adjustment for sex, race/ethnicity, age at cohort entry, highest level of education, disability, unemployment, household per capita income decile, number of family members per bedroom, family size, number of children in family, household flooring, household piped water access, quintiles of household expenditure on medicines, quintile of per capita household expenditure on food, formal labor employment, formal labor employment in the family, whether the family receives *Bolsa Familia* or not, and whether the individual has been hospitalized before FHS use. HRs for lengths of time since first observation are interpreted relative to those without any FHS use. FHS, Family Health Strategy; HR, hazard ratio; IPTW, inverse probability treatment weighting.

In IPTW-RA survival analysis, an FHS user had a lower hazard of all-cause death (HR 0.564, 95% CI 0.544–0.585, *p* < 0.001) compared with an FHS nonuser (Fig 1). The HR is interpreted relative to the mean time since first FHS use (2.6 years). The predicted 5-year risk of death was 19.78 per 1,000 individuals for FHS nonusers and 11.45 per 1,000 FHS users with a RD of 8.32 (95% CI 7.8–8.9, *p* < 0.001). The covariates in the adjusted models reveal patterns in the hazard of death across demographic and socioeconomic groups (S7 Table). The hazard of death was higher for individuals who were male (compared with female); who self-classified as black, *pardo*, or other racial groups (compared with white); who were disabled (compared with no reported disability); who were informally employed (compared with formally employed); who had any prior hospitalizations (compared with no hospitalizations); and who were in households receiving a conditional cash transfer (compared with those that did not). There was a gradient of increasing HRs across age groups and a decreasing gradient HRs across educational groups (the lowest HRs for those with the highest educational attainment), household income groups (lowest HRs for the highest income groups), and household food expenditure quintiles (lowest HRs for the highest expenditure group). Time- and utilization-varying analyses revealed the largest reductions in hazard of death were found for those who had been FHS users for at least 1 year (Fig 2) and who had 3 or more consultations over the period (see S8 Table and S4 Fig).

**Fig 3:**
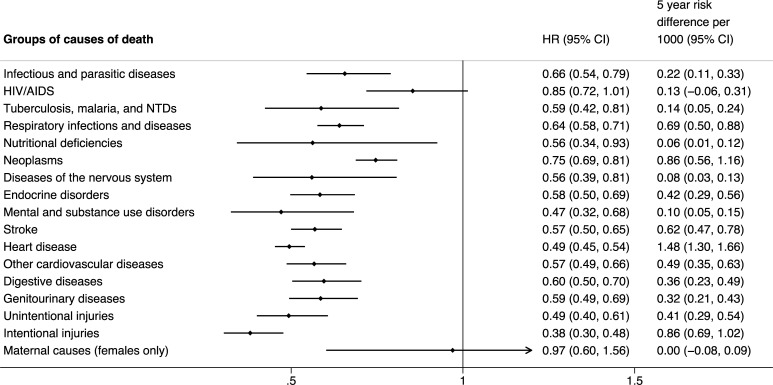
HRs from FHS usage on time until death from selected causes. Estimated HRs obtained from flexible parametric survival models with IPTW and regression adjustment for sex, race/ethnicity, age at cohort entry, highest level of education, disability, unemployment, household per capita income decile, number of family members per bedroom, family size, number of children in family, household flooring, household piped water access, quintiles of household expenditure on medicines, quintile of per capita household expenditure on food, formal labor employment, formal labor employment in the family, whether the family receives *Bolsa Familia* or not, and whether the individual has been hospitalized before FHS use. A separate model was carried out for each groups of causes. HRs interpreted relative to a mean time since FHS usage of 2.6 years. Infectious and parasitic diseases exclude HIV/AIDs, tuberculosis, malaria, and NTDs. FHS, Family Health Strategy; HR, hazard ratio; IPTW, inverse probability treatment weighting; NTD, neglected tropical disease.

By selected causes of death, FHS users had lower hazards of death all groups of causes except HIV/AIDS and maternal causes of death (Fig 3). The largest absolute reductions (5-year RD) were found for heart disease (RD 1.5 [95% CI 1.3–1.7, *p* < 0.001]), neoplasms (RD 0.9 [95% CI 0.6–1.2, *p* < 0.001]), intentional injuries (RD 0.9 [95% CI 0.7–1.0, *p* < 0.001]) and respiratory infections and diseases (RD 0.7 [95% CI 0.5–0.9, *p* < 0.001]). By detailed subgroup causes of death (see S3 Fig), a general pattern was evident in which FHS users had greater reductions in hazard of death for causes more amenable to healthcare (e.g., tuberculosis, intestinal infections, diabetes, cardiovascular diseases, and alcohol and drug use disorders) compared with causes less amenable to healthcare (e.g., uterine, mouth, and throat cancers; nervous system diseases; drownings; falls; and intentional self-harm) [31].

**Fig 4:**
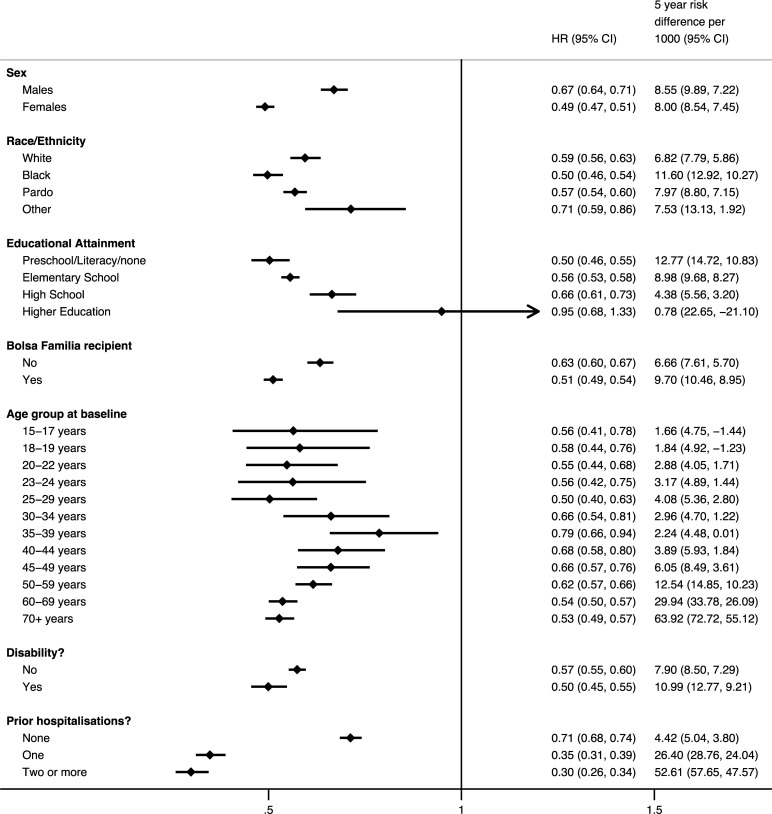
Subgroups analysis showing HRs and 5-year RDs from FHS usage on time until death from any cause. Estimated HRs and risk differences RDs obtained from flexible parametric survival models with IPTW and regression adjustment for sex, race/ethnicity, age at cohort entry, highest level of education, disability, unemployment, household per capita income decile, number of family members per bedroom, family size, number of children in family, household flooring, household piped water access, quintiles of household expenditure on medicines, quintile of per capita household expenditure on food, formal labor employment, formal labor employment in the family, whether the family receives *Bolsa Familia* or not, and whether the individual has been hospitalized before FHS use. A separate model was carried out for each subpopulation. Five-year RD estimated risk of death using mean survival curves. HRs and RD interpreted relative to a mean time since FHS usage of 2.6 years. FHS, Family Health Strategy; HR, hazard ratio; IPTW, inverse probability treatment weighting; RD, risk difference.

Exploring inequalities revealed heterogeneous associations from FHS use (Fig 4). FHS usage was associated with larger reductions in all-cause mortality for females (HR 0.49, 95% CI 0.47–0.51, *p* < 0.001) compared with males (HR 0.67, 95% CI 0.64–0.71, *p* < 0.001). However, because of higher underlying mortality rates for males, the 5-year RD was similar (approximately 8 per 1,000). Significant reductions for men in the hazard of death from FHS use were only found for cardiovascular disease, digestive diseases, renal and urinary diseases, and external causes of death (see S4 Fig).

By racial groups, reduction in the hazard of death for FHS users was largest for black Brazilians (HR 0.50 [95% CI 0.46–0.54, *p* < 0.001]); 5-year RD: 11.6 (95% CI 10.3–12.9, *p* < 0.001), compared with *pardo* (HR 0.57 [95% CI 0.54–0.60, *p* < 0.001]); 5-year RD: 8.0 (95% CI 7.2–8.8, *p* < 0.001) and white Brazilians (HR 0.59 [95% CI 0.56–0.63, *p* < 0.001]); 5-year RD: 6.8 (95% CI 5.9–7.8, *p* < 0.001). There were notable reductions in the hazard of death from HIV/AIDS, tuberculosis, and substance for black FHS users compared with smaller or nonsignificant reductions for white and *pardo* users (see S4 Fig).

By educational attainment, those with the lowest attainment (preschool, literacy class, or no education) had the greatest reductions in risk of hazard (HR 0.50 [95% CI 0.46–0.55, *p* < 0.001]); 5-year RD: 12.8 (95% CI 10.8–14.7, *p* < 0.001). This compared with those with elementary schooling (HR 0.56 (95% CI 0.53–0.58, *p* < 0.001); 5-year RD: 9.0 (95% CI 8.3–9.7, *p* < 0.001) or high-school education (HR 0.66 (95% CI 0.61–0.73, *p* < 0.001); 5-year RD: 4.4 (95% CI 3.2–5.6, *p* < 0.001). There was no significant association of FHS use for those with highest level of education (This gradient across educational groups was present for diabetes, cardiovascular disease, mental health and neurological disorders, respiratory infections and diseases, and external causes of death (see S4 Fig)).

For individuals in *Bolsa Familia–*receiving households, there were also greater associated reductions in the hazard of death from FHS use (HR 0.51 [95% CI 0.49–0.54, *p* < 0.001]); 5-year RD: 9.7 (95% CI 9.0–10.5, *p* < 0.001]) compared with nonrecipient households (HR 0.63 [95% CI 0.60–0.67, *p* < 0.001]); 5-year RD: 6.7 (95% CI 5.7–7.6, *p* < 0.001).

### Sensitivity analyses

Alternative model specifications and sequential additional of covariates demonstrate the robustness of the findings and model stability (see S4 Table, S5 Table, S6 Table and S5 Fig).

## Discussion

This study indicates that rapid expansion of the FHS in Rio de Janeiro was associated with substantial reductions in the risk of death in urban poor populations. Those with lower education, in *Bolsa Familia–*receiving households, or who were black or *pardo* (mixed-ethnicity) had greater relative and absolute reductions in the risk of death with evidence that the program reduced social gradients in mortality. The associated declined in mortality associated with PHC utilization was greater among those using services more frequently and over longer periods, increasing the plausibility of the findings. Furthermore, reductions in risk of death were generally greater for conditions in which PHC can exert the greater benefit through early detection, prevention, routine management, or timely referral to secondary or emergency care.

The finding that PHC is associated with health improvements is consistent with evidence from Brazil [11,13,14] and LMICs [32,33] and identified effect sizes for mortality similar with other Brazilian cohort studies [34]. PHC plausibly contributes to mortality reductions by increasing access to healthcare and improving quality of existing services, as prior to PHC expansion in the city, access for low-income populations was limited to overcrowded emergency rooms [15]. In addition to alleviating acute conditions and managing chronic diseases, PHC likely delivers health gains through preventive activities, health promotion, and community outreach [9]. The findings illustrate the greatest associated reductions in mortality accrue after 1 or 2 years of PHC use, which suggests services may be addressing unmet health needs.

The study’s individual-level dataset permits novel insights on the associations between the FHS and health gradients in urban poor populations [5]. Specifically, there was a hierarchical association of reductions in mortality across ethnic and education categories, where those with the greatest vulnerability having greater associated reductions in mortality most from FHS use. This finding is unsurprising, given black and *pardo* populations, those with lower educational attainment, or in receipt of conditional cash transfers have poorer health outcomes, higher rates of healthcare underutilization, higher levels of unhealthy behaviors, and exposed to structural discrimination and racism [35–37]. With poorer health outcomes and greater unmet needs, they are more likely to benefit more than other socioeconomic groups from increased access to healthcare. Although a universal approach was adopted, FHS expansion in Rio de Janeiro was initially prioritized to address large unmet health needs in underserved populations. The finding of synergistic health benefits between the FHS and *Bolsa Familia* is also concordant with other studies [11,38], likely stemming from conditionalities promoting healthcare usage and the known benefits from conditional cash transfer programs in addressing the social determinants of health and improving health outcomes [39]. Additionally, greater benefits from PHC use were identified among those with a prior hospitalization or a disability and also for diseases related to poverty (e.g., tuberculosis, HIV/AIDS, substance abuse, and mental health conditions). This aides an inequality-reducing interpretation of PHC but also highlights the importance of PHC in both primary and secondary prevention. International evidence also demonstrates PHC can contribute to more equitable health outcomes [40], and nationwide ecological studies from Brazil show inequality-reducing effects of the FHS [11,14,41].

This study also raises questions on populations not registered or using the FHS in Rio de Janeiro. Over 40% of the cohort (nearly 500,000 adults) were not registered with FHS services; an additional 275,000 were registered but were not users. Our study covered a period of FHS expansion when primary care clinics were either not rolled out to all parts of the city or were not reaching all individuals in their catchment areas. Beyond program rollout of clinics, other barriers to access may exist, including forgoing income to attend the clinic, transport costs, cultural considerations, perceived discrimination, or waiting times. Understanding the health outcomes and healthcare utilization patterns of these populations is a research priority, and further expanding access within the city should be a political priority.

There are key strengths to this study. It uses an extensive and large dataset of individual records with linked health and healthcare data. Linkage quality was high with a precision of 99%. In-depth analyses of socioeconomic differences are possible with detailed individual and household data which has been seldom possible in previous research. It also provides substantial statistical power to detect associations across small subgroups of causes. A key novelty is the focus on urban welfare-claiming families, which are often under-represented in other studies, are often treated as a homogenous “poor” population, and are absent from many studies on primary care in low- and middle-income countries (LMICs). Lastly, IPTW-RA was also employed as one of the strongest methods to minimize potential unobserved biases in observational studies [25].

However, there are key limitations. Systematic differences between registered/nonregistered individuals may exist, potentially introducing bias. Unobserved differences in underlying health status and propensity to seek healthcare may be present, potentially influencing likelihood of FHS use and risk of death. However, IPTW-RA was used to balance all observed covariates between FHS users and nonusers and to minimize the potential and impact of unobserved confounding [25]. The analysis also accounted for variable time since first FHS use addressing immortal time bias [26]. Additionally, FHS service rollout was not universal across the city, and some areas remained uncovered. As individuals could only register and use FHS services if they lived proximate to local clinics, there are no fees for accessing services, and there is limited ability to afford private health insurance, bias from selection into the program is minimized.

We identified a reduction in intentional injuries associated with FHS registration. Primary care services may play a role in reducing intentional violence by increasing identification of at-risk populations, offering emergency treatments, addressing risk factors such as mental health conditions and improved security around healthcare units [42]. However, this raises a question about whether the implementation of concurrent interventions may partly explain our findings. The rollout of the FHS in Rio de Janeiro coincided with implementation of a major violence pacification program and transport investments for the 2014 FIFA World Cup and 2016 Olympics in the city, which contributed to reduced violence, better socioeconomic and living standards, and changes in the urban environment [43–45]. However, these programs were not coordinated and were implemented separately, each in a phased manner, and would not explain the large reductions in mortality across a variety of conditions seen, including CVD.

The linkage of administrative datasets could also have introduced bias—especially if true record linkage was associated with FHS usage. This potential bias was minimized by separate linkage of *Cadastro Único* to mortality datasets and primary care records without either linkage affecting the other. Furthermore, evaluation of the linkages revealed a precision of 99%. Furthermore, any disparity in the 99% linkage precision between FHS usage groups is also unlikely to substantially undermine the findings. There may have been the potential for bias from excluding duplicate records, but these only account for 4.7% of the original cohort and are unlikely to substantially alter the findings.

This study provides valuable evidence on the importance of investing in PHC to improve health and reduce health inequalities in urban poor populations globally. These findings are likely generalizable to other low-income urban populations in LMICs. Strengthening PHC in poor urban environments remains a challenge to achieving UHC, including in slum populations, which are projected to reach two billion people globally by 2030 [40,46]. The recent financial crises in Brazil, which was felt most acutely in Rio de Janeiro, threatens PHC delivery because of substantial funding cuts. PHC has been further undermined by increases in gang-related violence in the city since the economic crisis in 2015. This underlines the importance of achieving and maintaining political support for investment in PHC, including embracing a model of universal provision around which popular support can be built—especially during periods when public expenditures are severely constrained. Other studies of PHC in Rio de Janeiro have identified long distances to clinics, wait times, staff and equipment shortages, and perceived poor treatment by healthcare professionals as barriers to accessing healthcare, and addressing these is essential to ensure investments in PHC that characterized the program in Rio de Janeiro are sustained [47].

## Conclusion

Expansion of FHS in the city of Rio de Janeiro since 2008 was associated with substantial health improvements and reductions in health inequalities in urban poor populations. Increased investment in PHC is likely to improve health and reduce health inequalities among vulnerable populations globally.

## Supporting information

S1 RECORD Checklist (DOCX)

S1 Fig FHS coverage in state capitals of Brazil 2002–2016. FHS, Family Health Strategy.
(DOCX)

S2 Fig Adjusted survival function of cohort by FHS usage groups based on a chronological analysis time. FHS, Family Health Strategy.
(DOCX)

S3 Fig Hazard ratios for FHS users (compared with nonusers) by selected causes of death. FHS, Family Health Strategy.
(DOCX)

S4 Fig Hazard ratios for FHS users (compared with nonusers) by selected causes of death across socioeconomic and demographic groups. FHS, Family Health Strategy.
(DOCX)

S5 Fig Hazard ratios for all-cause mortality and number of FHS by cumulative usage. FHS, Family Health Strategy.
(DOCX)

S1 Table Overview of data sources and variables. (DOCX)

S2 Table Groups of causes of death by ICD-10 codes. ICD-10, International Classification of Disease 10th edition.
(DOCX)

S3 Table Comparison of cohort characteristics for unweighted and IPTW with standardized differences. IPTW, inverse probabilities of treatment weighting.
(DOCX)

S4 Table Alternative distribution specifications of parametric and flexible parametric models. (DOCX)

S5 Table Survival analysis results with sequential addition of groups of covariates. (DOCX)

S6 Table Survival analysis models on FHS usage on all-cause mortality with alternative time, weighting, and regression adjustment specifications. FHS, Family Health Strategy.
(DOCX)

S7 Table Results from flexible proportional hazards model on time-to-death from any cause. (DOCX)

S8 Table Hazard ratios for all-cause mortality and number of FHS by cumulative usage. FHS, Family Health Strategy.
(DOCX)

S1 Text Methods for linking routine administrative datasets. (DOCX)

S2 Text Formula for calculating IPTW. IPTW, inverse probabilities of treatment weighting.
(DOCX)

## Abbreviations

CVD: cardiovascular disease
FHS: Family Health Strategy
IPTW-RA: inverse probability treatment weighting and regression adjustment
LMIC: low- and middle-income country
PHC: primary healthcare
RD: risk difference
UHC: universal health coverage
