# Supplementary material for: Primary healthcare expansion and mortality in Brazil’s urban poor: A cohort analysis of 1.2 million adults
Source: PLoS Med. 2020 Oct 30;17(10):e1003357. doi: 10.1371/journal.pmed.1003357 (PMC7598481; doi:10.1371/journal.pmed.1003357)
Supplement: S1 Fig — FHS, Family Health Strategy. (DOCX) [file pmed.1003357.s002.docx]

**S1 Fig. FHS coverage in state capitals of Brazil 2002-2016**


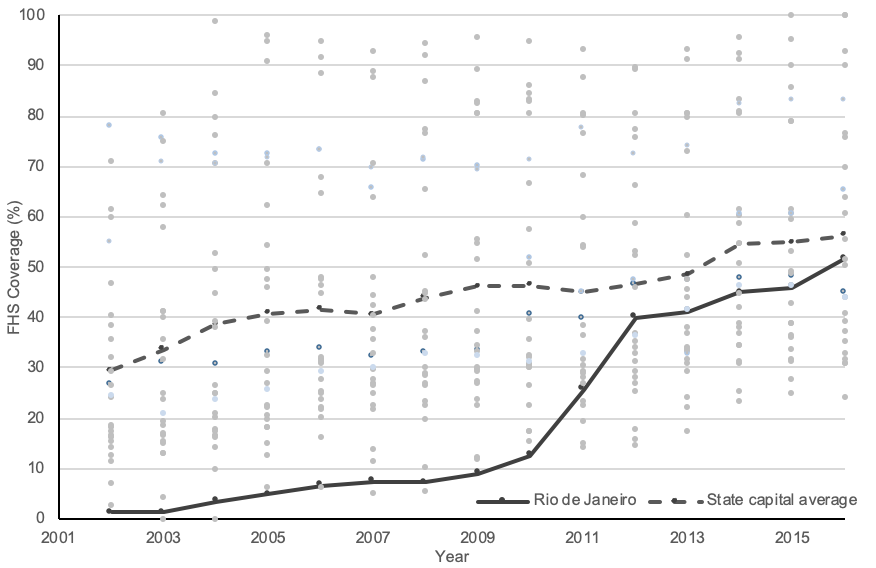


Source: Departamento de Atenção Básica (DAB) Ministério da Saúde. Histórico de Cobertura da Saúde da Família [Historical Coverage of the Family Health Programme]. 2020. <https://egestorab.saude.gov.br/paginas/acessoPublico/relatorios/relHistoricoCoberturaAB.xhtml>
