# Supplementary material for: Primary healthcare expansion and mortality in Brazil’s urban poor: A cohort analysis of 1.2 million adults
Source: PLoS Med. 2020 Oct 30;17(10):e1003357. doi: 10.1371/journal.pmed.1003357 (PMC7598481; doi:10.1371/journal.pmed.1003357)
Supplement: S2 Fig — FHS, Family Health Strategy. (DOCX) [file pmed.1003357.s003.docx]

**S2 Fig. Adjusted survival function of cohort by FHS usage groups based on a chronological analysis time**

Survival functions obtained from flexible parametric survival models using chronological age (at start of cohort) as analysis time and with IPTW and regression adjustment for: sex, race/ethnicity, age at cohort entry, highest level of education, disability, unemployment, household per capita income decile, number of family members per bedroom, family size, number of children in family, household flooring, household piped water access, quintiles of household expenditure on medicines, quintile of per capita household expenditure on food, formal labour employment, formal labour employment in the family, if the family receives Bolsa Familia or not, and if the individual has been hospitalised before FHS use.
