# Supplementary material for: Primary healthcare expansion and mortality in Brazil’s urban poor: A cohort analysis of 1.2 million adults
Source: PLoS Med. 2020 Oct 30;17(10):e1003357. doi: 10.1371/journal.pmed.1003357 (PMC7598481; doi:10.1371/journal.pmed.1003357)
Supplement: S3 Fig — FHS, Family Health Strategy. (DOCX) [file pmed.1003357.s004.docx]

**S3 Figure. Hazard ratios for FHS users (compared to non-users) by selected causes of death**
