# Supplementary material for: Primary healthcare expansion and mortality in Brazil’s urban poor: A cohort analysis of 1.2 million adults
Source: PLoS Med. 2020 Oct 30;17(10):e1003357. doi: 10.1371/journal.pmed.1003357 (PMC7598481; doi:10.1371/journal.pmed.1003357)
Supplement: S1 Table — (DOCX) [file pmed.1003357.s007.docx]

**S1 Table. Overview of data sources and variables**

| **Database** | **Overview** | **Source** | **Time period** | **Key variables (with categories)** |
| --- | --- | --- | --- | --- |
| Cadastro Único | A national administrative database on all individuals who are claiming government welfare. Contains 1.2 million adults aged 15 years or older. | *Secretaria Municipal de Assistência Social* (Municipal Secretariat of Social Assistance; SMAS)) | 2015 extraction (all individuals registered before 1^st^ January 2015) | **Individual-level variables**  Sex (male or female); race/ethnicity (black, white, mixed race (*pardo*), or other); age at cohort entry (15-17 years, 18-19, 20-22, 23-24, 25-29, 30-34, 35-39, 40-44, 45-49, 50-59, 60-69, and 70 years or more); highest level education (preschool/literacy/none, elementary school, high-school, or higher education); disability (yes or no); unemployed (yes or no); formal labour employment (yes or no);  **Household-level variables**  household per capita income decile; number of family members per bedroom (two or fewer, two-three, three-four, four or more); family size (one, two, three, four, five, six or more); number of children in family (one, two, three, four or more); household flooring (cement, wood, ceramic or tiles, or other); household piped water access (yes or no); quintiles of household expenditure on medicines; quintile of per capita household expenditure on food; formal labour employment in the family (yes or no); Bolsa Familia receiving family (yes or no). |
| ESF Electronic health records (EHR) | A municipal dataset containing dates of individuals’ registration with FHS clinics and the dates of their contacts with health professionals. | Secretaria Municipal de Saúde (Municipal Health Secretariat; SMS) | 1^st^ January 2010 to 31^st^ December 2016 | Date of FHS clinic registration; Date of FHS clinic utilisation. |
| Sistema de Informações sobre Mortalidade (the Mortality Information System; SIM) | National universal dataset of all death certificates | SMS | 1^st^ January 2000 to 31^st^ December 2016 | Date of death; cause of death (ICD-10 code). |
| Sistema de Informações Hospitalares  (Hospitalisation Information System; SIH) | National dataset of all hospital records for the public health system. | SMS | 1^st^ January 2000 to 31^st^ December 2016 | Total hospitalisations prior to FHS use (none, one, two or more times). |
