# Supplementary material for: Primary healthcare expansion and mortality in Brazil’s urban poor: A cohort analysis of 1.2 million adults
Source: PLoS Med. 2020 Oct 30;17(10):e1003357. doi: 10.1371/journal.pmed.1003357 (PMC7598481; doi:10.1371/journal.pmed.1003357)
Supplement: S2 Table — ICD-10, International Classification of Disease 10th edition. (DOCX) [file pmed.1003357.s008.docx]

**S2 Table. Groups of causes of death by International Classification of Disease (ICD-10) codes**

| Group of causes | ICD10 Codes |
| --- | --- |
| Infectious and parasitic diseases (excluding HIV/aids, Tuberculosis, malaria, and neglected tropical diseases) | A00-B99, G00-G04, G14, N70-N73, P37.3, P37.4  (Except B20-B24, A15-A19, B90, B50-B54, P37.3, P37.4, A30, A31.1, A66, A71, A82, A90-A91, A92.0, B47, B55-B57, B65, B66.3-B66.4, B66.0-B66.1, B67, B68, B69, B72, B73 B74.0-B74.2, B76.0-B76.1, B77, B79) |
| HIV/aids | B20-B24 |
| Tuberculosis, malaria, and neglected tropical diseases | A15-A19, B90, B50-B54, P37.3, P37.4, A30, A31.1, A66, A71, A82, A90-A91, A92.0, B47, B55-B57, B65, B66.3-B66.4, B66.0-B66.1, B67, B68, B69, B72, B73 B74.0-B74.2, B76.0-B76.1, B77, B79 |
| Respiratory infections and diseases | H65-H66, J00- J98, P23, U04 |
| Nutritional deficiencies | D50-D53, D64.9, E00-E02, E40-E46, E50-E64 |
| Neoplasms | C00-D48 |
| Diseases of the nervous system | F00-F03.9, G10-G13.8, G20-G21.0, G21.2-G24, G24-G25.0, G25.2-G25.3, G25.5, G25.8-G26.0, G30-G31.1, G31.8-G31.9, G35-G37.9, G40-G41.9, G61-G61.9, G70-G72, G72.2-G73.7, G90-G90.9, G95-G95.9, M33-M33.9 |
| Endocrine disorders | D55-D89 (minus D64.9), E03-E07, E10-E14 (minus E10.2-E10.29, E11.2-E11.29, E12.2, E13.2-E13.29, E14.2), E15-E34, E65-E88 |
| Mental and substance use disorders | F04-F99, G72.1, Q86.0, X41-X42, X44, X45 |
| Stroke | I60-I69.9 |
| Heart disease | I20-I59.9 |
| Other cardiovascular diseases | I00-I09.9 I10-5.9 I70-I99.9 |
| Digestive diseases | K20-K92.9 |
| Genitourinary diseases | E10.2-E10.29,E11.2-E11.29,E12.2,E13.2-E13.29,E14.2, N00-N64, N75-N76, N80-N98 |
| Unintentional injuries | V01-X40, X43, X46-59, Y40-Y86, Y88, Y89 |
| Intentional injuries | X60-Y09, Y35-Y36, Y870, Y871 |
| Maternal causes (females only) | O00-O99 |
|  |  |

Source: WHO Global Observatory for Health R&D <http://www.who.int/research-observatory/en/>
