# Supplementary material for: Primary healthcare expansion and mortality in Brazil’s urban poor: A cohort analysis of 1.2 million adults
Source: PLoS Med. 2020 Oct 30;17(10):e1003357. doi: 10.1371/journal.pmed.1003357 (PMC7598481; doi:10.1371/journal.pmed.1003357)
Supplement: S3 Table — IPTW, inverse probabilities of treatment weighting. (DOCX) [file pmed.1003357.s009.docx]

**S3 Table. Comparison of cohort characteristics for unweighted and IPTW with standardised differences**

|  |  | Unweighted Distribution (%) | | |  | IPTW Distribution (%) | | |
| --- | --- | --- | --- | --- | --- | --- | --- | --- |
|  |  | Non-FHS users | FHS users | Std Diff |  | Non-FHS users | FHS users | Std Diff |
| Individual characteristics |  |  |  |  |  |  |  |  |
| Sex |  |  |  |  |  |  |  |  |
| Male |  | 29.9 | 45.2 | -0.321 |  | 39.5 | 39.4 | 0.001 |
| Female |  | 70.1 | 54.8 | 0.321 |  | 60.5 | 60.6 | -0.001 |
| Race |  |  |  |  |  |  |  |  |
| White |  | 29.0 | 29.9 | -0.018 |  | 29.7 | 29.6 | 0.002 |
| Black |  | 17.8 | 17.2 | 0.017 |  | 17.4 | 17.4 | 0.000 |
| Parda (mixed) |  | 51.2 | 50.5 | 0.014 |  | 50.6 | 50.7 | -0.002 |
| Other |  | 1.9 | 2.4 | -0.035 |  | 2.3 | 2.3 | 0.001 |
| Age (years) |  |  |  |  |  |  |  |  |
| 15-17 |  | 9.8 | 11.2 | -0.045 |  | 10.9 | 10.7 | 0.005 |
| 18-19 |  | 6.4 | 7.2 | -0.032 |  | 6.8 | 6.8 | -0.001 |
| 20-22 |  | 8.6 | 10.2 | -0.056 |  | 9.4 | 9.5 | -0.003 |
| 23-24 |  | 4.9 | 6.2 | -0.055 |  | 5.6 | 5.7 | -0.003 |
| 25-29 |  | 9.7 | 11.6 | -0.062 |  | 10.7 | 10.8 | -0.004 |
| 30-34 |  | 8.9 | 9.2 | -0.011 |  | 9.0 | 9.1 | -0.003 |
| 35-39 |  | 9.4 | 9.2 | 0.006 |  | 9.2 | 9.3 | -0.001 |
| 40-44 |  | 8.8 | 8.4 | 0.017 |  | 8.6 | 8.5 | 0.001 |
| 45-49 |  | 8.0 | 7.2 | 0.032 |  | 7.6 | 7.5 | 0.002 |
| 50-59 |  | 13.4 | 10.5 | 0.089 |  | 11.8 | 11.7 | 0.004 |
| 60-69 |  | 8.3 | 5.7 | 0.102 |  | 6.8 | 6.8 | 0.002 |
| 70+ |  | 3.8 | 3.5 | 0.016 |  | 3.7 | 3.6 | 0.002 |
| Education level |  |  |  |  |  |  |  |  |
| Preschool/Literacy class/None |  | 7.5 | 8.8 | -0.047 |  | 8.3 | 8.3 | 0.001 |
| Elementary school |  | 62.4 | 60.2 | 0.045 |  | 60.9 | 61.0 | -0.002 |
| High school |  | 29.0 | 29.2 | -0.004 |  | 29.2 | 29.2 | 0.001 |
| Higher education |  | 1.0 | 1.8 | -0.063 |  | 1.5 | 1.5 | 0.002 |
| Disability |  |  |  |  |  |  |  |  |
| No |  | 95.2 | 96.8 | -0.082 |  | 96.1 | 96.1 | -0.001 |
| Yes |  | 4.8 | 3.2 | 0.082 |  | 3.9 | 3.9 | 0.001 |
| Unemployed |  |  |  |  |  |  |  |  |
| No |  | 69.2 | 76.5 | -0.164 |  | 73.7 | 73.7 | -0.001 |
| Yes |  | 30.8 | 23.5 | 0.164 |  | 26.3 | 26.3 | 0.001 |
| Formally employed? |  |  |  |  |  |  |  |  |
| No |  | 91.3 | 91.3 | -0.001 |  | 91.3 | 91.3 | 0.000 |
| Yes |  | 8.7 | 8.7 | 0.001 |  | 8.7 | 8.7 | 0.000 |
| Hospitalisations prior to FHS |  |  |  |  |  |  |  |  |
| None |  | 89.4 | 86.2 | 0.097 |  | 87.0 | 87.3 | -0.008 |
| one |  | 8.1 | 9.4 | -0.049 |  | 9.8 | 8.6 | 0.042 |
| two or more |  | 2.6 | 4.4 | -0.098 |  | 3.1 | 4.1 | -0.051 |
| Household characteristics |  |  |  |  |  |  |  |  |
| Income Deciles |  |  |  |  |  |  |  |  |
| Q1 (poorest) |  | 8.5 | 10.2 | -0.062 |  | 9.5 | 9.6 | -0.002 |
| Q2 |  | 9.2 | 8.8 | 0.015 |  | 8.8 | 8.9 | -0.003 |
| Q3 |  | 9.6 | 9.0 | 0.021 |  | 9.1 | 9.2 | -0.002 |
| Q4 |  | 9.7 | 9.3 | 0.016 |  | 9.4 | 9.4 | -0.001 |
| Q5 |  | 10.0 | 9.8 | 0.007 |  | 9.8 | 9.8 | 0.000 |
| Q6 |  | 9.9 | 9.8 | 0.005 |  | 9.8 | 9.8 | 0.000 |
| Q7 |  | 10.1 | 9.9 | 0.010 |  | 10.0 | 10.0 | 0.001 |
| Q8 |  | 10.5 | 10.1 | 0.013 |  | 10.3 | 10.3 | 0.002 |
| Q9 |  | 10.8 | 10.7 | 0.000 |  | 10.9 | 10.8 | 0.003 |
| Q10 (richest) |  | 11.8 | 12.5 | -0.023 |  | 12.4 | 12.3 | 0.003 |
| Family members per bedroom |  |  |  |  |  |  |  |  |
| 2 or fewer |  | 41.9 | 38.9 | 0.059 |  | 40.2 | 40.1 | 0.001 |
| more than 2, 3 or fewer |  | 25.3 | 25.0 | 0.006 |  | 25.1 | 25.1 | 0.000 |
| more than 3, 4 or fewer |  | 17.3 | 18.3 | -0.026 |  | 18.0 | 17.9 | 0.000 |
| greater than 4 |  | 15.5 | 17.7 | -0.059 |  | 16.7 | 16.8 | -0.002 |
| Family size |  |  |  |  |  |  |  |  |
| Single person |  | 6.4 | 6.4 | -0.001 |  | 6.4 | 6.4 | 0.001 |
| Two |  | 13.9 | 12.6 | 0.037 |  | 13.2 | 13.1 | 0.001 |
| Three |  | 20.4 | 20.3 | 0.004 |  | 20.4 | 20.3 | 0.001 |
| Four |  | 22.7 | 23.2 | -0.011 |  | 23.0 | 23.0 | 0.001 |
| Five |  | 16.5 | 16.8 | -0.010 |  | 16.7 | 16.7 | -0.001 |
| Six or more |  | 20.2 | 20.7 | -0.014 |  | 20.3 | 20.4 | -0.003 |
| Number of children in family |  |  |  |  |  |  |  |  |
| None |  | 49.1 | 53.0 | -0.078 |  | 51.9 | 51.6 | 0.004 |
| One |  | 29.6 | 28.4 | 0.026 |  | 28.8 | 28.9 | -0.002 |
| Two |  | 14.4 | 12.8 | 0.048 |  | 13.3 | 13.3 | -0.002 |
| Three |  | 4.9 | 4.2 | 0.036 |  | 4.4 | 4.4 | -0.002 |
| Four or more |  | 2.0 | 1.6 | 0.024 |  | 1.7 | 1.7 | -0.001 |
| Household flooring material |  |  |  |  |  |  |  |  |
| Soil |  | 19.4 | 26.6 | -0.171 |  | 23.9 | 23.9 | 0.000 |
| Cement |  | 20.4 | 17.2 | 0.081 |  | 18.3 | 18.4 | -0.002 |
| Re-purposed wood |  | 1.7 | 2.0 | -0.025 |  | 1.9 | 1.9 | 0.001 |
| Ceramics/tiles |  | 57.0 | 52.3 | 0.095 |  | 54.1 | 54.1 | 0.001 |
| Other |  | 1.5 | 1.9 | -0.029 |  | 1.8 | 1.8 | 0.000 |
| Piped water in household? |  |  |  |  |  |  |  |  |
| No |  | 2.5 | 3.0 | -0.034 |  | 2.8 | 2.8 | -0.001 |
| Yes |  | 97.5 | 97.0 | 0.034 |  | 97.2 | 97.2 | 0.001 |
| Bolsa Familia claiming family? |  |  |  |  |  |  |  |  |
| No |  | 28.9 | 36.0 | -0.152 |  | 33.6 | 33.4 | 0.004 |
| Yes |  | 71.1 | 64.0 | 0.152 |  | 66.4 | 66.6 | -0.004 |
| Quintiles of per capita medicine expenditure |  |  |  |  |  |  |  |  |
| Q1 (least) |  | 79.0 | 80.8 | -0.044 |  | 80.0 | 80.1 | -0.001 |
| Q2 |  | 8.1 | 6.9 | 0.047 |  | 7.3 | 7.3 | -0.001 |
| Q3 |  | 5.5 | 5.1 | 0.017 |  | 5.3 | 5.3 | 0.000 |
| Q4 |  | 4.0 | 3.7 | 0.016 |  | 3.8 | 3.8 | 0.001 |
| Q5 (most) |  | 3.4 | 3.6 | -0.008 |  | 3.6 | 3.5 | 0.002 |
| Formal employment in family |  |  |  |  |  |  |  |  |
| No |  | 78.6 | 78.4 | 0.004 |  | 78.5 | 78.5 | -0.001 |
| Yes |  | 21.4 | 21.6 | -0.004 |  | 21.5 | 21.5 | 0.001 |
| Quintiles of per capita food expenditure |  |  |  |  |  |  |  |  |
| Q1 (least) |  | 20.1 | 23.1 | -0.071 |  | 21.9 | 21.9 | -0.001 |
| Q2 |  | 20.5 | 18.6 | 0.049 |  | 19.2 | 19.3 | -0.002 |
| Q3 |  | 20.7 | 19.8 | 0.024 |  | 20.1 | 20.1 | -0.001 |
| Q4 |  | 20.0 | 19.9 | 0.003 |  | 20.0 | 19.9 | 0.001 |
| Q5 (most) |  | 18.7 | 18.7 | -0.001 |  | 18.8 | 18.7 | 0.002 |

FHS – family health strategy; Std Diff – standardised difference.
