# Supplementary material for: Primary healthcare expansion and mortality in Brazil’s urban poor: A cohort analysis of 1.2 million adults
Source: PLoS Med. 2020 Oct 30;17(10):e1003357. doi: 10.1371/journal.pmed.1003357 (PMC7598481; doi:10.1371/journal.pmed.1003357)
Supplement: S4 Table — (DOCX) [file pmed.1003357.s010.docx]

**S4 Table. Alternative distribution specifications of parametric and flexible parametric models**

| **Distribution** | **Log Likelihood** | **AIC** | **BIC** |
| --- | --- | --- | --- |
| **Parametric models** |  |  |  |
| Exponential | -132780.8 | 265611.7 | 265912.5 |
| Weibull | -132546.5 | 265145.1 | 265457.9 |
| Gompertz | -132387.8 | 264827.6 | 265140.4 |
| Lognormal | -133232.8 | 266517.7 | 266830.5 |
| Loglogistic | -132546.8 | 265145.6 | 265458.5 |
| **Flexible parametric models** |  |  |  |
| PH (1) | -132547 | 265145.1 | 265457.9 |
| PH (2) | -132428 | 264910.6 | 265235.4 |
| PH (3) | -132375 | 264805.2 | 265142.0 |
| PH (4) | -132369 | 264796.8 | 265145.7 |
| PH (5) | -132361 | 264782.2 | 265143.2 |
| PH (6) | -132360 | 264781.5 | 265154.5 |
| PH (7) | -132361 | 264785.3 | 265170.3 |

AIC - Akaike information criterion; BIC - Bayesian information criterion; PH – Proportional Hazards model. Model include FHS utilisation, sex, race/ethnicity, age group, and income quintiles as covariates. Number if brackets for Flexible parametric models refers to degrees of freedom for modelling the distribution function.

AIC values suggest a flexible proportional hazard model with 6 degrees of freedom offers the best fit for the survival function. However, this is very close to the Gompertz distribution in a parametric model.

Source: Royston P, Lambert PC. Flexible Parametric Survival Analysis Using Stata: Beyond the Cox Model: Stata Press; 2011.
