# Supplementary material for: Primary healthcare expansion and mortality in Brazil’s urban poor: A cohort analysis of 1.2 million adults
Source: PLoS Med. 2020 Oct 30;17(10):e1003357. doi: 10.1371/journal.pmed.1003357 (PMC7598481; doi:10.1371/journal.pmed.1003357)
Supplement: S5 Table — (DOCX) [file pmed.1003357.s011.docx]

**S5 Table. Survival analysis results with sequential addition of groups of covariates.**

|  | Model 1 | Model 2 | Model 3 | Model 4 | Model 5 | Model 6 | Model 7 | Model 8 | Model 9 | Model 10 |
| --- | --- | --- | --- | --- | --- | --- | --- | --- | --- | --- |
|  | **HR** | **HR** | **HR** | **HR** | **HR** | **HR** | **HR** | **HR** | **HR** | **HR** |
|  | **(95%CI)** | **(95%CI)** | **(95%CI)** | **(95%CI)** | **(95%CI)** | **(95%CI)** | **(95%CI)** | **(95%CI)** | **(95%CI)** | **(95%CI)** |
| FHS Usage |  |  |  |  |  |  |  |  |  |  |
| No (Ref) | 1 | 1 | 1 | 1 | 1 | 1 | 1 | 1 | 1 | 1 |
| Yes | 0.588 | 0.595 | 0.507 | 0.508 | 0.510 | 0.512 | 0.511 | 0.512 | 0.512 | 0.564 |
|  | 0.568,0.610 | 0.574,0.616 | 0.490,0.526 | 0.490,0.526 | 0.493,0.529 | 0.494,0.530 | 0.493,0.530 | 0.494,0.531 | 0.494,0.530 | 0.544,0.585 |
| Sex |  |  |  |  |  |  |  |  |  |  |
| Male (Ref) | - | 1 | 1 | 1 | 1 | 1 | 1 | 1 | 1 | 1 |
| Female | - | 0.669 | 0.619 | 0.61 | 0.601 | 0.602 | 0.602 | 0.598 | 0.597 | 0.582 |
|  |  | 0.654,0.684 | 0.605,0.633 | 0.597,0.624 | 0.588,0.615 | 0.588,0.616 | 0.588,0.616 | 0.585,0.612 | 0.584,0.611 | 0.568,0.595 |
| Race/ethnicity |  |  |  |  |  |  |  |  |  |  |
| White (Ref) | - | 1 | 1 | 1 | 1 | 1 | 1 | 1 | 1 | 1 |
| Black | - | 1.300 | 1.388 | 1.395 | 1.37 | 1.354 | 1.337 | 1.339 | 1.344 | 1.328 |
|  |  | 1.260,1.341 | 1.345,1.432 | 1.351,1.439 | 1.328,1.414 | 1.312,1.397 | 1.295,1.380 | 1.297,1.382 | 1.302,1.387 | 1.286,1.372 |
| Parda | - | 0.898 | 1.088 | 1.093 | 1.083 | 1.079 | 1.076 | 1.079 | 1.081 | 1.09 |
|  |  | 0.874,0.922 | 1.059,1.118 | 1.064,1.123 | 1.055,1.113 | 1.050,1.109 | 1.048,1.106 | 1.050,1.109 | 1.053,1.111 | 1.061,1.121 |
| Other | - | 1.564 | 1.453 | 1.46 | 1.475 | 1.489 | 1.482 | 1.501 | 1.511 | 1.468 |
|  |  | 1.463,1.672 | 1.356,1.557 | 1.363,1.563 | 1.377,1.580 | 1.390,1.595 | 1.383,1.588 | 1.401,1.608 | 1.410,1.619 | 1.368,1.574 |
| Age (years) |  |  |  |  |  |  |  |  |  |  |
| 15-17 (Ref) | - | - | 1 | 1 | 1 | 1 | 1 | 1 | 1 | 1 |
| 18-19 | - | - | 1.498 | 1.488 | 1.478 | 1.47 | 1.452 | 1.454 | 1.46 | 1.377 |
|  |  |  | 1.286,1.745 | 1.277,1.733 | 1.269,1.722 | 1.262,1.713 | 1.247,1.692 | 1.248,1.694 | 1.253,1.701 | 1.182,1.605 |
| 20-22 | - | - | 1.829 | 1.768 | 1.745 | 1.725 | 1.664 | 1.674 | 1.719 | 1.478 |
|  |  |  | 1.594,2.099 | 1.540,2.029 | 1.520,2.003 | 1.503,1.981 | 1.449,1.910 | 1.458,1.922 | 1.498,1.974 | 1.287,1.697 |
| 23-24 | - | - | 1.933 | 1.848 | 1.834 | 1.81 | 1.719 | 1.737 | 1.822 | 1.488 |
|  |  |  | 1.676,2.229 | 1.602,2.132 | 1.590,2.115 | 1.569,2.088 | 1.490,1.984 | 1.505,2.004 | 1.579,2.103 | 1.289,1.717 |
| 25-29 | - | - | 2.045 | 1.954 | 1.938 | 1.91 | 1.824 | 1.843 | 1.939 | 1.567 |
|  |  |  | 1.787,2.341 | 1.707,2.238 | 1.693,2.219 | 1.668,2.187 | 1.593,2.089 | 1.610,2.111 | 1.693,2.221 | 1.368,1.795 |
| 30-34 | - | - | 2.06 | 1.938 | 1.927 | 1.922 | 1.85 | 1.867 | 1.996 | 1.669 |
|  |  |  | 1.792,2.369 | 1.685,2.228 | 1.675,2.215 | 1.671,2.211 | 1.608,2.128 | 1.623,2.148 | 1.735,2.297 | 1.450,1.921 |
| 35-39 | - | - | 2.375 | 2.223 | 2.229 | 2.243 | 2.182 | 2.201 | 2.377 | 2.11 |
|  |  |  | 2.071,2.724 | 1.938,2.550 | 1.943,2.557 | 1.955,2.573 | 1.901,2.503 | 1.918,2.525 | 2.071,2.728 | 1.837,2.422 |
| 40-44 | - | - | 2.838 | 2.657 | 2.688 | 2.693 | 2.619 | 2.644 | 2.856 | 2.67 |
|  |  |  | 2.480,3.248 | 2.322,3.042 | 2.348,3.076 | 2.353,3.082 | 2.288,2.998 | 2.310,3.026 | 2.495,3.270 | 2.332,3.057 |
| 45-49 | - | - | 4.162 | 3.872 | 3.943 | 3.926 | 3.795 | 3.834 | 4.131 | 4.003 |
|  |  |  | 3.646,4.752 | 3.391,4.422 | 3.453,4.503 | 3.438,4.483 | 3.324,4.334 | 3.357,4.378 | 3.617,4.719 | 3.505,4.573 |
| 50-59 | - | - | 8.175 | 7.41 | 7.598 | 7.473 | 7.188 | 7.265 | 7.783 | 7.411 |
|  |  |  | 7.196,9.288 | 6.521,8.421 | 6.686,8.634 | 6.576,8.492 | 6.324,8.170 | 6.392,8.258 | 6.846,8.848 | 6.518,8.425 |
| 60-69 | - | - | 18.214 | 15.598 | 16.165 | 15.594 | 14.965 | 15.058 | 15.864 | 14.293 |
|  |  |  | 16.039,20.684 | 13.725,17.726 | 14.222,18.373 | 13.717,17.728 | 13.161,17.016 | 13.243,17.122 | 13.950,18.041 | 12.567,16.256 |
| 70+ | - | - | 36.269 | 30.765 | 33.945 | 32.662 | 30.801 | 30.800 | 31.920 | 27.74 |
|  |  |  | 31.935,41.191 | 27.054,34.984 | 29.829,38.629 | 28.694,37.178 | 27.050,35.074 | 27.044,35.078 | 28.024,36.357 | 24.353,31.599 |
| Education level |  |  |  |  |  |  |  |  |  |  |
| Preschool/Literacy class/None (Ref) | - | - | 1 | 1 | 1 | 1 | 1 | 1 | 1 | 1 |
| Elementary school | - | - | 0.858 | 0.849 | 0.881 | 0.882 | 0.894 | 0.891 | 0.898 | 0.892 |
|  |  |  | 0.830,0.887 | 0.821,0.877 | 0.852,0.911 | 0.853,0.912 | 0.865,0.925 | 0.861,0.921 | 0.868,0.929 | 0.862,0.923 |
| High school | - | - | 0.537 | 0.528 | 0.567 | 0.572 | 0.59 | 0.587 | 0.6 | 0.609 |
|  |  |  | 0.514,0.562 | 0.506,0.552 | 0.542,0.592 | 0.547,0.598 | 0.564,0.617 | 0.561,0.614 | 0.574,0.628 | 0.582,0.637 |
| Higher education | - | - | 0.532 | 0.522 | 0.571 | 0.567 | 0.577 | 0.565 | 0.578 | 0.613 |
|  |  |  | 0.471,0.600 | 0.463,0.589 | 0.506,0.645 | 0.502,0.639 | 0.511,0.652 | 0.501,0.638 | 0.512,0.652 | 0.543,0.694 |
| Disability |  |  |  |  |  |  |  |  |  |  |
| No (Ref) | - | - | - | 1 | 1 | 1 | 1 | 1 | 1 | 1 |
| Yes | - | - | - | 1.786 | 1.82 | 1.783 | 1.794 | 1.753 | 1.752 | 1.478 |
|  |  |  |  | 1.718,1.856 | 1.751,1.892 | 1.714,1.854 | 1.725,1.867 | 1.684,1.824 | 1.684,1.824 | 1.418,1.540 |
| Unemployed |  |  |  |  |  |  |  |  |  |  |
| No (Ref) | - | - | - | 1 | 1 | 1 | 1 | 1 | 1 | 1 |
| Yes | - | - | - | 1.19 | 1.212 | 1.197 | 1.279 | 1.266 | 1.167 | 1.084 |
|  |  |  |  | 1.160,1.221 | 1.181,1.245 | 1.166,1.230 | 1.243,1.316 | 1.230,1.302 | 1.133,1.202 | 1.052,1.116 |
| Income Deciles |  |  |  |  |  |  |  |  |  |  |
| Q1 (poorest) (Ref) | - | - | - | - | 1 | 1 | 1 | 1 | 1 | 1 |
| Q2 | - | - | - | - | 0.859 | 0.868 | 0.884 | 0.883 | 0.878 | 0.896 |
|  |  |  |  |  | 0.816,0.905 | 0.824,0.914 | 0.839,0.931 | 0.839,0.930 | 0.834,0.925 | 0.851,0.944 |
| Q3 | - | - | - | - | 0.806 | 0.817 | 0.835 | 0.833 | 0.83 | 0.859 |
|  |  |  |  |  | 0.766,0.848 | 0.776,0.860 | 0.794,0.879 | 0.792,0.877 | 0.788,0.873 | 0.816,0.905 |
| Q4 | - | - | - | - | 0.762 | 0.777 | 0.799 | 0.799 | 0.795 | 0.826 |
|  |  |  |  |  | 0.724,0.803 | 0.738,0.819 | 0.758,0.842 | 0.758,0.842 | 0.754,0.838 | 0.783,0.871 |
| Q5 | - | - | - | - | 0.659 | 0.682 | 0.699 | 0.705 | 0.704 | 0.746 |
|  |  |  |  |  | 0.625,0.694 | 0.647,0.719 | 0.663,0.737 | 0.668,0.743 | 0.668,0.743 | 0.706,0.787 |
| Q6 | - | - | - | - | 0.646 | 0.668 | 0.687 | 0.692 | 0.695 | 0.749 |
|  |  |  |  |  | 0.613,0.681 | 0.634,0.704 | 0.652,0.724 | 0.657,0.730 | 0.659,0.733 | 0.710,0.791 |
| Q7 | - | - | - | - | 0.599 | 0.623 | 0.647 | 0.649 | 0.659 | 0.718 |
|  |  |  |  |  | 0.569,0.632 | 0.591,0.657 | 0.613,0.682 | 0.615,0.684 | 0.625,0.695 | 0.680,0.759 |
| Q8 | - | - | - | - | 0.59 | 0.62 | 0.646 | 0.653 | 0.67 | 0.726 |
|  |  |  |  |  | 0.560,0.621 | 0.589,0.652 | 0.613,0.680 | 0.620,0.688 | 0.636,0.706 | 0.688,0.766 |
| Q9 | - | - | - | - | 0.563 | 0.594 | 0.625 | 0.636 | 0.662 | 0.713 |
|  |  |  |  |  | 0.536,0.591 | 0.566,0.623 | 0.595,0.656 | 0.605,0.669 | 0.629,0.696 | 0.676,0.751 |
| Q10 (richest) | - | - | - | - | 0.555 | 0.551 | 0.591 | 0.604 | 0.635 | 0.692 |
|  |  |  |  |  | 0.530,0.581 | 0.527,0.577 | 0.564,0.619 | 0.575,0.635 | 0.604,0.668 | 0.656,0.730 |
| Family members per bedroom |  |  |  |  |  |  |  |  |  |  |
| 2 or fewer | - | - | - | - | 1 | 1 | 1 | 1 | 1 | 1 |
| more than 2, 3 or fewer | - | - | - | - | 0.879 | 0.932 | 0.895 | 0.901 | 0.904 | 0.906 |
|  |  |  |  |  | 0.851,0.907 | 0.898,0.967 | 0.862,0.930 | 0.867,0.935 | 0.870,0.939 | 0.873,0.941 |
| more than 3, 4 or fewer | - | - | - | - | 0.825 | 0.898 | 0.832 | 0.842 | 0.846 | 0.839 |
|  |  |  |  |  | 0.795,0.856 | 0.860,0.937 | 0.795,0.870 | 0.805,0.880 | 0.808,0.885 | 0.802,0.878 |
| greater than 4 | - | - | - | - | 0.889 | 0.85 | 0.752 | 0.763 | 0.774 | 0.765 |
|  |  |  |  |  | 0.857,0.922 | 0.813,0.889 | 0.717,0.790 | 0.727,0.801 | 0.738,0.813 | 0.728,0.803 |
| Family size |  |  |  |  |  |  |  |  |  |  |
| Single person | - | - | - | - | - | 1 | 1 | 1 | 1 | 1 |
| Two | - | - | - | - | - | 0.831 | 0.841 | 0.832 | 0.828 | 0.867 |
|  |  |  |  |  |  | 0.799,0.864 | 0.808,0.875 | 0.799,0.866 | 0.796,0.862 | 0.832,0.903 |
| Three | - | - | - | - | - | 0.752 | 0.771 | 0.761 | 0.755 | 0.815 |
|  |  |  |  |  |  | 0.717,0.789 | 0.734,0.809 | 0.725,0.799 | 0.719,0.793 | 0.775,0.857 |
| Four | - | - | - | - | - | 0.724 | 0.756 | 0.741 | 0.732 | 0.817 |
|  |  |  |  |  |  | 0.689,0.761 | 0.719,0.796 | 0.705,0.779 | 0.696,0.770 | 0.776,0.861 |
| Five | - | - | - | - | - | 0.81 | 0.866 | 0.842 | 0.826 | 0.936 |
|  |  |  |  |  |  | 0.769,0.854 | 0.822,0.913 | 0.798,0.887 | 0.783,0.871 | 0.886,0.989 |
| Six or more | - | - | - | - | - | 0.944 | 1.012 | 0.977 | 0.953 | 1.095 |
|  |  |  |  |  |  | 0.897,0.995 | 0.960,1.066 | 0.927,1.030 | 0.903,1.006 | 1.036,1.157 |
| Number of children in family |  |  |  |  |  |  |  |  |  |  |
| None | - | - | - | - | - | 1 | 1 | 1 | 1 | 1 |
| One | - | - | - | - | - | 0.944 | 0.981 | 0.973 | 0.978 | 0.96 |
|  |  |  |  |  |  | 0.913,0.975 | 0.948,1.014 | 0.940,1.006 | 0.945,1.011 | 0.928,0.993 |
| Two | - | - | - | - | - | 0.996 | 1.055 | 1.045 | 1.053 | 0.995 |
|  |  |  |  |  |  | 0.951,1.042 | 1.007,1.106 | 0.997,1.095 | 1.005,1.104 | 0.949,1.043 |
| Three | - | - | - | - | - | 0.975 | 1.047 | 1.038 | 1.048 | 0.941 |
|  |  |  |  |  |  | 0.908,1.047 | 0.974,1.126 | 0.965,1.116 | 0.975,1.127 | 0.875,1.012 |
| Four or more | - | - | - | - | - | 1.039 | 1.132 | 1.122 | 1.129 | 0.939 |
|  |  |  |  |  |  | 0.937,1.153 | 1.020,1.258 | 1.010,1.246 | 1.017,1.254 | 0.845,1.044 |
| Household flooring material |  |  |  |  |  |  |  |  |  |  |
| Soil | - | - | - | - | - | - | 1 | 1 | 1 | 1 |
| Cement | - | - | - | - | - | - | 0.939 | 0.911 | 0.96 | 1.012 |
|  |  |  |  |  |  |  | 0.903,0.977 | 0.875,0.948 | 0.922,1.000 | 0.971,1.055 |
| Re-purposed wood | - | - | - | - | - | - | 0.847 | 0.823 | 0.87 | 0.911 |
|  |  |  |  |  |  |  | 0.783,0.917 | 0.760,0.891 | 0.803,0.942 | 0.840,0.989 |
| Cermanics/tiles | - | - | - | - | - | - | 0.769 | 0.745 | 0.788 | 0.838 |
|  |  |  |  |  |  |  | 0.742,0.796 | 0.719,0.772 | 0.760,0.818 | 0.807,0.870 |
| Other | - | - | - | - | - | - | 0.997 | 0.979 | 1.043 | 1.042 |
|  |  |  |  |  |  |  | 0.919,1.082 | 0.902,1.062 | 0.961,1.132 | 0.958,1.134 |
| Piped water in household? |  |  |  |  |  |  |  |  |  |  |
| No (Ref) | - | - | - | - | - | - | 1 | 1 | 1 | 1 |
| Yes | - | - | - | - | - | - | 0.935 | 0.936 | 0.932 | 0.934 |
|  |  |  |  |  |  |  | 0.877,0.996 | 0.878,0.997 | 0.875,0.994 | 0.876,0.996 |
| Bolsa Familia claiming family? |  |  |  |  |  |  |  |  |  |  |
| No (Ref) | - | - | - | - | - | - | - | 1 | 1 | 1 |
| Yes | - | - | - | - | - | - | - | 1.125 | 1.127 | 1.1 |
|  |  |  |  |  |  |  |  | 1.093,1.159 | 1.094,1.161 | 1.068,1.134 |
| Quintiles of per capita medicine expenditure |  |  |  |  |  |  |  |  |  |  |
| Q1 (least) (Ref) | - | - | - | - | - | - | - | 1 | 1 | 1 |
| Q2 | - | - | - | - | - | - | - | 0.971 | 0.968 | 0.975 |
|  |  |  |  |  |  |  |  | 0.927,1.018 | 0.924,1.015 | 0.930,1.022 |
| Q3 | - | - | - | - | - | - | - | 1.016 | 1.014 | 1.017 |
|  |  |  |  |  |  |  |  | 0.968,1.066 | 0.966,1.064 | 0.969,1.068 |
| Q4 | - | - | - | - | - | - | - | 1.108 | 1.106 | 1.097 |
|  |  |  |  |  |  |  |  | 1.053,1.166 | 1.051,1.163 | 1.042,1.155 |
| Q5 (most) | - | - | - | - | - | - | - | 1.281 | 1.275 | 1.276 |
|  |  |  |  |  |  |  |  | 1.224,1.341 | 1.219,1.334 | 1.218,1.337 |
| Formal employment in family |  |  |  |  |  |  |  |  |  |  |
| No (Ref) | - | - | - | - | - | - | - | - | 1 | 1 |
| Yes | - | - | - | - | - | - | - | - | 1.047 | 1.083 |
|  |  |  |  |  |  |  |  |  | 1.005,1.091 | 1.039,1.129 |
| Formally employed? |  |  |  |  |  |  |  |  |  |  |
| No (Ref) | - | - | - | - | - | - | - | - | 1 | 1 |
| Yes | - | - | - | - | - | - | - | - | 0.549 | 0.547 |
|  |  |  |  |  |  |  |  |  | 0.510,0.592 | 0.508,0.589 |
| Quintiles of per capita food expenditure |  |  |  |  |  |  |  |  |  |  |
| Q1 (least) (Ref) | - | - | - | - | - | - | - | - | - | 1 |
| Q2 | - | - | - | - | - | - | - | - | - | 0.878 |
|  |  |  |  |  |  |  |  |  |  | 0.848,0.910 |
| Q3 | - | - | - | - | - | - | - | - | - | 0.868 |
|  |  |  |  |  |  |  |  |  |  | 0.837,0.900 |
| Q4 | - | - | - | - | - | - | - | - | - | 0.861 |
|  |  |  |  |  |  |  |  |  |  | 0.828,0.894 |
| Q5 (most) | - | - | - | - | - | - | - | - | - | 0.757 |
|  |  |  |  |  |  |  |  |  |  | 0.725,0.789 |
| Hospitalisations prior to FHS |  |  |  |  |  |  |  |  |  |  |
| None (Ref) | - | - | - | - | - | - | - | - | - | 1 |
| One | - | - | - | - | - | - | - | - | - | 3.016 |
|  |  |  |  |  |  |  |  |  |  | 2.926,3.109 |
| Two or more | - | - | - | - | - | - | - | - | - | 5.741 |
|  |  |  |  |  |  |  |  |  |  | 5.556,5.932 |
|  |  |  |  |  |  |  |  |  |  |  |
| AIC | 301231 | 299348 | 262725 | 261499 | 260468 | 260147 | 259845 | 259682 | 259379 | 248282 |
| BIC | 301330 | 299496 | 263045 | 261844 | 260961 | 260750 | 260509 | 260408 | 260130 | 249107 |

Notes: AIC - Akaike information criterion; BIC - Bayesian information criterion; FHS – Family Health Strategy; HR – Hazard Ratio; CI – Confidence interval.
