# Supplementary material for: Primary healthcare expansion and mortality in Brazil’s urban poor: A cohort analysis of 1.2 million adults
Source: PLoS Med. 2020 Oct 30;17(10):e1003357. doi: 10.1371/journal.pmed.1003357 (PMC7598481; doi:10.1371/journal.pmed.1003357)
Supplement: S6 Table — FHS, Family Health Strategy. (DOCX) [file pmed.1003357.s012.docx]

**S6 Table. Survival analysis models on FHS usage on all-cause mortality with alternative time, weighting, and regression adjustment specifications**

|  | **M1 (Main model IPTW-RA)** | **M2 (chronological time scale)** | **M3 (RA only)** | **M4 (IPTW only)** | **M5 (IPTW on FHS registration)** | **M6 (IPTW on family member FHS registration)** | **M7 (unadjusted and unweighted)** |
| --- | --- | --- | --- | --- | --- | --- | --- |
| **IPTW** |  |  |  |  |  |  |  |
| FHS usage | Y | Y | - | Y | - | - | - |
| Individual registration with FHS | - | - | - | - | Y | - | - |
| Family member registration with FHS | - | - | - | - | - | Y | - |
|  |  |  |  |  |  |  |  |
| **RA** | Y | Y | Y | - | Y | Y | - |
|  |  |  |  |  |  |  |  |
|  |  |  |  |  |  |  |  |
| **Time scale** |  |  |  |  |  |  |  |
| Chronological age | - | Y | - | - | - | - | - |
| Time-on-study | Y | - | Y | Y | Y | Y | Y |
|  |  |  |  |  |  |  |  |
| FHS usage (HR) | 0.564 | 0.614 | 0.523 | 0.588 | 0.494 | 0.479 | 0.521 |
| 95% CI | 0.544,0.585 | 0.593,0.636 | 0.505,0.541 | 0.568,0.610 | 0.478,0.512 | 0.463,0.496 | 0.503,0.539 |

Notes: FHS – Family Health Strategy; HR – Hazard Ratio; IPTW – Inverse Probability of Treatment Weighting; RA – Regression Adjustment; CI – Confidence interval.
