# Supplementary material for: Primary healthcare expansion and mortality in Brazil’s urban poor: A cohort analysis of 1.2 million adults
Source: PLoS Med. 2020 Oct 30;17(10):e1003357. doi: 10.1371/journal.pmed.1003357 (PMC7598481; doi:10.1371/journal.pmed.1003357)
Supplement: S7 Table — (DOCX) [file pmed.1003357.s013.docx]

**S7 Table. Results from flexible proportional hazards model on time to death from any cause**

|  | **HR** | **95%CI** |
| --- | --- | --- |
| ESF Usage |  |  |
| No (Ref) | 1 (ref) |  |
| Yes | 0.564 | [0.544,0.585] |
| **Individual-level characteristics** |  |  |
| Sex |  |  |
| Male (Ref) | 1 (ref) |  |
| Female | 0.582 | [0.568,0.595] |
| Race/ethnicity |  |  |
| White (Ref) | 1 (ref) |  |
| Black | 1.328 | [1.286,1.372] |
| Parda | 1.090 | [1.061,1.121] |
| Other | 1.468 | [1.368,1.574] |
| Age (years) |  |  |
| 15-17 (Ref) | 1 (ref) |  |
| 18-19 | 1.377 | [1.182,1.605] |
| 20-22 | 1.478 | [1.287,1.697] |
| 23-24 | 1.488 | [1.289,1.717] |
| 25-29 | 1.567 | [1.368,1.795] |
| 30-34 | 1.669 | [1.450,1.921] |
| 35-39 | 2.110 | [1.837,2.422] |
| 40-44 | 2.670 | [2.332,3.057] |
| 45-49 | 4.003 | [3.505,4.573] |
| 50-59 | 7.411 | [6.518,8.425] |
| 60-69 | 14.293 | [12.567,16.256] |
| 70+ | 27.740 | [24.353,31.599] |
| Education level |  |  |
| Preschool/Literacy class/None (Ref) | 1 (ref) |  |
| Elementary school | 0.892 | [0.862,0.923] |
| High school | 0.609 | [0.582,0.637] |
| Higher education | 0.613 | [0.543,0.694] |
| Disability |  |  |
| No (Ref) | 1 (ref) |  |
| Yes | 1.478 | [1.418,1.540] |
| Unemployed |  |  |
| No (Ref) | 1 (ref) |  |
| Yes | 1.084 | [1.052,1.116] |
| Formally employed? |  |  |
| No (Ref) | 1 (ref) |  |
| Yes | 0.547 | [0.508,0.589] |
| Hospitalisations prior to ESF |  |  |
| None (Ref) | 1 (ref) |  |
| one | 3.016 | [2.926,3.109] |
| two or more | 5.741 | [5.556,5.932] |
| **Household-level characteristics** |  |  |
| Income Deciles |  |  |
| Q1 (poorest) (Ref) | 1 (ref) |  |
| Q2 | 0.896 | [0.851,0.944] |
| Q3 | 0.859 | [0.816,0.905] |
| Q4 | 0.826 | [0.783,0.871] |
| Q5 | 0.746 | [0.706,0.787] |
| Q6 | 0.749 | [0.710,0.791] |
| Q7 | 0.718 | [0.680,0.759] |
| Q8 | 0.726 | [0.688,0.766] |
| Q9 | 0.713 | [0.676,0.751] |
| Q10 (richest) | 0.692 | [0.656,0.730] |
| Family members per bedroom |  |  |
| 2 or fewer | 1 (ref) |  |
| more than 2, 3 or fewer | 0.906 | [0.873,0.941] |
| more than 3, 4 or fewer | 0.839 | [0.802,0.878] |
| greater than 4 | 0.765 | [0.728,0.803] |
| Family size |  |  |
| Single person | 1 (ref) |  |
| Two | 0.867 | [0.832,0.903] |
| Three | 0.815 | [0.775,0.857] |
| Four | 0.817 | [0.776,0.861] |
| Five | 0.936 | [0.886,0.989] |
| Six or more | 1.095 | [1.036,1.157] |
| Number of children in family |  |  |
| None | 1 (ref) |  |
| One | 0.960 | [0.928,0.993] |
| Two | 0.995 | [0.949,1.043] |
| Three | 0.941 | [0.875,1.012] |
| Four or more | 0.939 | [0.845,1.044] |
| Household flooring material |  |  |
| Soil | 1 (ref) |  |
| Cement | 1.012 | [0.971,1.055] |
| Re-purposed wood | 0.911 | [0.840,0.989] |
| Cermanics/tiles | 0.838 | [0.807,0.870] |
| Other | 1.042 | [0.958,1.134] |
| Piped water in household? |  |  |
| No (Ref) | 1 (ref) |  |
| Yes | 0.934 | [0.876,0.996] |
| Bolsa Familia claiming family? |  |  |
| No (Ref) | 1 (ref) |  |
| Yes | 1.100 | [1.068,1.134] |
| Quintiles of per capita medicine expenditure |  |  |
| Q1 (least) (Ref) | 1 (ref) |  |
| Q2 | 1.538 | [1.530,1.547] |
| Q3 | 0.969 | [0.966,0.971] |
| Q4 | 0.977 | [0.975,0.979] |
| Q5 (most) | 0.996 | [0.994,0.997] |
| Formal employment in family |  |  |
| No (Ref) | 1 (ref) |  |
| Yes | 1.083 | [1.039,1.129] |
| Quintiles of per capita food expenditure |  |  |
| Q1 (least) (Ref) | 1 (ref) |  |
| Q2 | 0.878 | [0.848,0.910] |
| Q3 | 0.868 | [0.837,0.900] |
| Q4 | 0.861 | [0.828,0.894] |
| Q5 (most) | 0.757 | [0.725,0.789] |
