# Supplementary material for: Primary healthcare expansion and mortality in Brazil’s urban poor: A cohort analysis of 1.2 million adults
Source: PLoS Med. 2020 Oct 30;17(10):e1003357. doi: 10.1371/journal.pmed.1003357 (PMC7598481; doi:10.1371/journal.pmed.1003357)
Supplement: S8 Table — FHS, Family Health Strategy. (DOCX) [file pmed.1003357.s014.docx]

**S8 Table. Hazard Ratios for all-cause mortality and number of FHS by cumulative usage**

| **Cumulative FHS usage** | **HR** | **95%CI** |
| --- | --- | --- |
| No FHS use (ref) | 1 (Ref) | - |
| 1 | 1.74 | 1.61, 1.89 |
| 2 | 1.04 | 0.94, 1.15 |
| 3 | 0.90 | 0.81 1.00 |
| 4 | 0.64 | 0.57, 0.73 |
| 5 | 0.66 | 0.58, 0.75 |
| 6-7 | 0.51 | 0.46, 0.57 |
| 8-9 | 0.48 | 0.43, 0.54 |
| 10-14 | 0.38 | 0.34, 0.41 |
| 15-19 | 0.29 | 0.25, 0.34 |
| 20-29 | 0.22 | 0.19, 0.25 |
| 30-49 | 0.19 | 0.15, 0.25 |
| 50+ | 0.156 | 0.11, 0.23 |

Estimated hazard ratios (HR) obtained from flexible parametric survival model with IPTW and regression adjustment for: sex, race/ethnicity, age at cohort entry, highest level of education, disability, unemployment, household per capita income decile, number of family members per bedroom, family size, number of children in family, household flooring, household piped water access, quintiles of household expenditure on medicines, quintile of per capita household expenditure on food, formal labour employment, formal labour employment in the family, if the family receives Bolsa Familia or not, and if the individual has been hospitalised before FHS use. Total FHS consultations calculated over the period 2010-2016, and individuals divided into groups based on their usage patterns: no FHS use; one consultation; two; three; four; five six-seven; eight-nine; 10-14; 15-19; 20-29; 30-49; or 50 or more consultations. These usage groups used as the variable of interest and HR estimated for each category of usage. HRs are interpreted relative to those without any FHS use.
