# Supplementary material for: Primary healthcare expansion and mortality in Brazil’s urban poor: A cohort analysis of 1.2 million adults
Source: PLoS Med. 2020 Oct 30;17(10):e1003357. doi: 10.1371/journal.pmed.1003357 (PMC7598481; doi:10.1371/journal.pmed.1003357)
Supplement: S1 Text — (DOCX) [file pmed.1003357.s015.docx]

**S1 Text. Methods for linking routine administrative datasets**

PostgreSQL and OpenReclink software were used to pre-process and link databases used in this analysis (see Gilmore and Treat 2006, and Camargo and Coeli 2015). A multiple linkage strategy was used combining deterministic, multiple-pass probabilistic, and extensive clerical review approaches. A sequential procedure was adopted where only records where no match was identified from the deterministic phase were sent to probabilistic linkage. Likewise, records were only sent to the subsequent probabilistic pass where no match was found in a previous pass.

For the deterministic approach, record pairs were classified as matches based on the exact agreement on the personal identifier (the CPF number (*Cadastro de Pessoas Físicas;* Brazilian tax number)) or a deterministic linkage key. The deterministic linkage key was created by concatenating the soundex phonetic code of the first given name, the soundex phonetic code of the second segment of the name (i.e. the second double given name or first family name), the soundex phonetic code of the last family name, the sex, and the date of birth (Christen 2015).

For the probabilistic linkage, a seven-pass blocking strategy was applied using indexing keys formed by different combinations of the following attributes: soundex phonetic code of the first name, soundex phonetic code of the last name, year of birth, and sex. Records were paired by name, mother's name, and date of birth, except for the sixth pass, where the name was replaced by the given name and the last family name, and the seventh pass, where the mother's name was omitted. The linkage weights were estimated through the Expectation-Maximization (EM) algorithm with a composite weight upper threshold defined empirically in each blocking pass (Herzog, Scheuren ans Winkler 2007). Levenshtein edit distance was used to compare names, and an exact character-by-character algorithm to compare the date of birth (Christen 2015). Candidate record pairs generated in the six first blocking steps that presented a composite weight equal to or higher than the upper threshold were classified as matches. In the seventh blocking pass, candidate record pairs were classified as potential matches and sent to be manually reviewed.

Eight reviewers manually assessed all candidate record pairs classified as potential matches. The reviewers were trained and certified by one research expert in clerical review, who was also responsible for their supervision. The rules for record pairs classification were based on all attributes used in the probabilistic process in addition to the address.

References

Gilmore WJ, Treat RH. Beginning PHP and PostgreSQL 8. New York: Apress, 2006.

Camargo Jr KR de, Coeli CM. Going open source: some lessons learned from the development of OpenRecLink. Cad Saúde Pública. 2015 Feb;31(2):257–63.

Christen P. Data matching concepts and techniques for record linkage, entity resolution, and duplicate detection. Berlin; New York: Springer; 2012.

Herzog T, Scheuren FJ, Winkler WE.Data Quality and Record Linkage Techniques. Berlin; New York: Springer; 2007.
