# Supplementary material for: Primary healthcare expansion and mortality in Brazil’s urban poor: A cohort analysis of 1.2 million adults
Source: PLoS Med. 2020 Oct 30;17(10):e1003357. doi: 10.1371/journal.pmed.1003357 (PMC7598481; doi:10.1371/journal.pmed.1003357)
Supplement: S2 Text — IPTW, inverse probabilities of treatment weighting. (DOCX) [file pmed.1003357.s016.docx]

**S2 Text. Formula for calculating inverse probabilities of treatment weighting (IPTW)**

Predicted probabilities were estimated from adjusted logistic regression on likelihood of FHS use. The IPTW was estimated from this predicted probability using the equation:

$$IPTW= \frac{Z}{e}+\frac{1-Z}{1-e}$$

Where *Z* refers to a binary variable denoting treatment (FHS usage; either 0 or 1), and *e* refers to the predicted probability of treatment (FHS usage) from the logistic regression model. The estimated IPTW were normalised to maintain the original sample size.

Source: Austin PC, Stuart EA. Moving towards best practice when using inverse probability of treatment weighting (IPTW) using the propensity score to estimate causal treatment effects in observational studies. *Statistics in medicine* 2015; **34**(28): 3661-79.
